# Physical Training and Pulmonary Rehabilitation in Patients with Cystic Fibrosis: A Systematic Review and Meta-Analysis of Clinical Trials

**DOI:** 10.3390/healthcare13162017

**Published:** 2025-08-15

**Authors:** Saray Ríos Murillo, Angie Melissa Hinestroza Mancilla, Lina Manuela Pérez Ordoñez, Naudy Yulisa Ararat Carabalí, Freiser Eceomo Cruz Mosquera, Yamil Liscano

**Affiliations:** Grupo de Investigación en Salud Integral (GISI), Department of Health Sciences Faculty, Universidad Santiago de Cali, Cali 760035, Colombia; sarayrios00@usc.edu.co (S.R.M.); angie.hinestroza00@usc.edu.co (A.M.H.M.); lina.perez15@usc.edu.co (L.M.P.O.); naudy.ararart00@usc.edu.co (N.Y.A.C.); yamil.liscano00@usc.edu.co (Y.L.)

**Keywords:** pulmonary rehabilitation, exercise therapy, cystic fibrosis, exercise, forced expiratory volume, quality of life

## Abstract

Background/Objectives: Physical training and Pulmonary Rehabilitation in Patients with Cystic Fibrosis: A Systematic Review and Meta-Analysis of Clinical Trials. Pulmonary rehabilitation and physical training are essential components of the comprehensive management of patients with cystic fibrosis. Despite documented benefits for some clinical outcomes, uncertainty exists regarding their overall effects. Therefore, the objective of the present meta-analysis is to determine the effectiveness of physical training and pulmonary rehabilitation in patients with CF. Methods: This systematic review and meta-analysis of randomized controlled trials published between 1990 and 2025 was conducted using the PubMed, Cochrane Clinical Trial, SCOPUS, Science Direct, Web of Science, Scielo, and LILAC databases. The risk of bias was evaluated using the RoB 2 tool, the quality of the evidence with the Jadad scale, and the certainty of the evidence for each outcome was assessed according to GRADE guidelines. This meta-analysis was developed using the statistical packages RevMan 5.4^®^ and Jamovi 2.3.28^®^. Results: Twenty-three studies with a total of 800 patients with CF were included. This meta-analysis showed that pulmonary rehabilitation and physical training did not affect pulmonary function, as observed in FEV1 (SMD: 0.05; 95% CI: −0.09 to 0.20; *p* = 0.46) and FVC (SMD: 0.11; 95% CI: −0.04 to 0.27; *p* = 0.14). However, it has a discrete impact on exercise capacity, producing an increase in VO_2_ max (MD: 2.74; 95% CI: 0.43 to 5.04; *p* = 0.02). Subgroup analyses did not yield relevant findings, and sensitivity analyses did not produce modifications in the direction or magnitude of the effect. Conclusions: The intervention evaluated in this meta-analysis does not have effects on pulmonary function but may influence exercise capacity, particularly VO_2_ max. It is recommended to interpret the findings with caution due to the limited certainty of the available evidence.

## 1. Introduction

Cystic fibrosis (CF) is a rare autosomal recessive genetic disease that affects multiple organ systems, with the respiratory, cardiovascular, and musculoskeletal systems being the most compromised [1,2]. CF originates from mutations in the cystic fibrosis transmembrane conductance regulator (CFTR) gene, located on chromosome 7 [3,4]. To date, more than 2100 variants in the CFTR gene have been described; however, approximately 719 have been shown to cause cystic fibrosis [5], as they lead to a significant decrease in or complete absence of CFTR protein function, a condition essential for the clinical manifestation of the disease [6,7,8]. These mutations variously alter the function of the CFTR protein, which is crucial for the proper transport of ions such as chloride, sodium, and water across cell membranes. Consequently, thick, dehydrated secretions occur, particularly in the lungs, leading to obstruction of glandular ducts [9,10,11].

Globally, CF affects approximately 105,000 individuals, involving diverse racial and ethnic populations [12]. It has been documented that this condition is more frequent in individuals of Caucasian descent, with an approximate incidence of 1 in 2500 live births [13,14]. In Europe, CF incidence ranges from 1 in 4500 to 1 in 6000 live births, depending on geographical area [15,16]. In North America, it is estimated that CF occurs in 1 in 3300 live births in Canada, similar to the slightly lower incidence of 1 in 4000 births reported in the United States [17,18]. In Latin America, the reported incidence of cystic fibrosis varies widely depending on the country and the availability of diagnostic resources. For instance, in Argentina, it is estimated at 1 in 1600 live births, whereas in Brazil, it is approximately 1 in 14,000 [19].

Although CF remains a serious condition, advances in diagnosis, treatment, and comprehensive management have allowed many patients, in the past three decades, to transition from a limited life expectancy to a manageable chronic condition, with substantial improvements in functionality, autonomy, health-related quality of life, and life expectancy [4,20]. With the sustained increase in the median age of the cystic fibrosis population and the consequent growth in the number of adults living with the disease, associated complications have become progressively more frequent [21,22]. Among these, the progressive decline in pulmonary function stands out, continuing to be one of the main causes of morbidity in these patients [23].

Currently, the treatment of patients with CF is primarily based on the use of certain drugs aimed at modulating the CFTR protein, improving pulmonary function, and controlling respiratory infections [24,25]. However, the therapeutic approach for these patients also includes complementary non-pharmacological care, such as nutritional monitoring, education, structured physical training, and pulmonary rehabilitation, which is defined as a multidisciplinary program that integrates supervised physical exercise, airway clearance techniques, respiratory training, and patient counseling [26,27,28]. The latter two have gained increasing relevance due to their potential impact on exercise tolerance, respiratory function, quality of life, and the reduction in exacerbations [29,30]. A study by Schmidt et al. [31], evaluated a 12-week aerobic training program in 42 patients with CF and observed improvements in functional capacity and some domains of quality of life. Similarly, Hulzebos et al. [32] reported that a six-week high-intensity interval training program in an adolescent with cystic fibrosis led to significant improvements in exercise capacity. Along the same lines, Gagulic et al. [33], in a group of 46 children with cystic fibrosis, evaluated the efficacy of the “KidMove” program, a home-based individualized physical exercise intervention supervised by parents. The study reported improvements in the distance covered in the incremental shuttle walk test, postural control, and emotional functioning. These findings are consistent with those reported by García et al. [34], who conducted a systematic review with a meta-analysis and found that physical exercise interventions in people with CF improve muscle strength, cardiovascular capacity, and respiratory muscle function.

Although there is a general consensus that interventions based on rehabilitation and physical training can offer benefits for people with cystic fibrosis, the current literature shows notable heterogeneity regarding the clinical outcomes evaluated and the characteristics of the applied interventions. Important gaps in knowledge persist concerning the magnitude and consistency of the effect of these interventions on key functional indicators, such as forced vital capacity and even the distance covered in the six-minute walk test. Additionally, it is not clearly established whether variables such as the type of exercise, its intensity, frequency, duration, or the context of application (hospital, outpatient, or home-based) modify the observed effects. This lack of clarity has limited the ability to establish definitive clinical recommendations based on consistent evidence. In this context, a rigorous quantitative synthesis of the existing evidence is essential. Therefore, this systematic review with meta-analysis is proposed to answer the following research question: in patients with cystic fibrosis (P), is pulmonary rehabilitation or physical training (I), compared with standard care—including pharmacological treatment, nutritional support, and routine clinical follow-up—C), effective for improving pulmonary function, improving exercise capacity, reducing pulmonary exacerbations, reducing hospitalizations, improving health-related quality of life, and reducing adverse effects (O)?

## 2. Materials and Methods

### 2.1. Study Protocol

We conducted this systematic review following the methodological guidance of the Cochrane Handbook and the PRISMA statement [35]. We registered the protocol a priori in the PROSPERO international prospective register of systematic reviews (CRD420251009437) and employed the PICO (Population, Intervention, Comparison, Outcomes) strategy to formulate our research question.

### 2.2. Eligibility Criteria

Inclusion Criteria

- Randomized controlled trials (RCTs), regardless of their specific design (e.g., crossover, parallel).
- Studies published in any language.
- Studies published between January 1990 and January 2025 were included to ensure a broad and representative evidence base from the period following the identification of the CFTR gene, as well as the evolution of exercise interventions in line with contemporary standards of care in cystic fibrosis.
- Pediatric and adult patients with a diagnosis of cystic fibrosis, enrolled in pulmonary rehabilitation or physical training programs of any modality (in-person or remote), regardless of intensity and setting (hospital, community center, or home).
- Studies reporting at least one of the following outcomes: pulmonary function (FEV1, FVC, FEV1/FVC, RV/TLC), exercise capacity (6MWT, VO_2_ max, Wmax), pulmonary exacerbations, hospitalizations, health-related quality of life, and adverse effects.

Exclusion Criteria

- Article preprints and letters to the editor.
- Studies published as conference abstracts.
- Studies not available in accessible formats.
- Patients with cardiac, orthopedic, or traumatic complications that prevent exercise performance or adequate participation in the rehabilitation program.
- Studies reporting on the same patient cohort as previous publications of similar research.

### 2.3. Data Sources and Search Strategy

The search was conducted in the following databases and search engines: PubMed, Cochrane Clinical Trial, SCOPUS, Science Direct, Web of Science, Scielo, and LILAC. Filters were applied when necessary to identify clinical trials, and the date range was set between 1990 and 2024. The search strategy was designed and executed from February to March 2025 (Search was last conducted on 28 March 2025) by two independent researchers (A.M.H.M., N.Y.A.C.) using the following keywords: Pulmonary Rehabilitation, Exercise Training, Respiratory Rehabilitation, and cystic fibrosis. The terms were combined using the Boolean operators AND and OR. The search strategy is described as follows: (“Cystic Fibrosis”) AND (“Pulmonary Rehabilitation” OR “Exercise therapy” OR “Respiratory physiotherapy”) AND (“Effectiveness” OR “Treatment outcome” OR “Pulmonary function” OR “Quality of life”). Additional web searches and manual reference checks were performed. When it was necessary to confirm critical trial data, ClinicalTrials.gov (https://clinicaltrials.gov/ (accessed on 2 April 2025)) or the repository indicated by the authors was accessed for verification. Rayyan—Intelligent Systematic Review (https://www.rayyan.ai/ (accessed on 10 April 2025)) and Zotero version 6.0 (accessed on 10 April 2025) were used for data storage.

### 2.4. Study Selection and Data Extraction

Two reviewers (L.M.P.O., A.M.H.M.) independently screened titles, abstracts, and subsequently full texts. Disagreements on inclusion were resolved by consensus or, if necessary, by a third reviewer. We contacted corresponding authors for inaccessible articles. Inter-rater reliability was measured with Cohen’s Kappa (κ). Subsequently, three reviewers (F.E.C.M., S.R.M., Y.L.) extracted data on study details (author, year, country), participant characteristics (*n*, population, age, sex), and intervention parameters (session/program duration, activities, frequency, setting). The primary outcomes extracted included measures of pulmonary function (FEV1, FVC, FEV1/FVC, RV/TLC), exercise capacity (6MWT, VO_2_ max, Wmax), pulmonary exacerbations, hospitalizations, health-related quality of life, and adverse effects. To ensure data integrity, a separate team of three reviewers (L.M.P.O., N.Y.A.C., and A.M.H.M.) then verified the completeness and accuracy of all recorded information.

### 2.5. Risk of Bias Assessment

The risk of bias assessment in the clinical trials was performed independently by reviewers F.E.C.M. and S.M.R. using a standardized (ROB 2.0) tool that incorporates the main methodological elements relevant to these studies [36]. The data obtained in the process were recorded in Review Manager software version 5.4^®^ (RevMan) (accessed on 24 April 2025). Various aspects were examined, including (a) method of random sequence generation, (b) concealment of participant allocation, (c) blinding of personnel and study subjects, (d) implementation of blinding in the assessment of outcomes, (e) completeness of the reported data, and (f) existence of bias in the presentation or reporting of findings.

Each criterion was classified into three categories: low, unclear, or high risk of bias, according to compliance with pre-established standards. Any divergence in the assessment of bias was resolved through discussion between the reviewers until consensus was reached.

### 2.6. Assessment of Evidence Quality

To evaluate the quality of the studies included in the meta-analysis, the Jadad scale was used. This scale provides an overall score from 0 to 5, with higher values indicating greater methodological quality. The scale is based on five fundamental criteria: (a) whether the study was randomized, (b) whether the intervention was implemented in a double-blind design, (c) whether participant withdrawals were recorded and described, (d) whether randomization was carried out using an appropriate method, and (e) whether the inclusion and exclusion criteria were clearly specified. Each criterion received a score of 0 for absence or insufficient description and 1 when present and correctly detailed. Clinical trials with a score between 0 and 2 were considered of low quality, while those with a score of 3 or more were classified as adequate quality. Although the Jadad scale assessment did not determine the exclusion of studies, its scores were considered in the analysis of the results.

### 2.7. Statistical Analysis

We performed the meta-analysis in RevMan 5.4 (accessed on 30 April 2025), calculating effect sizes and 95% CIs. An outcome was pooled only if reported by at least two studies. For continuous data, we extracted means and SDs (transforming medians/IQRs where necessary) and used the final time-point measurement. Effects were calculated as mean difference (MD) or standardized mean difference (SMD).

Heterogeneity was evaluated with the I^2^ statistic, guiding the choice of a fixed-effect (I^2^ < 50%) or random-effects (I^2^ ≥ 50%) model, both using the inverse variance method. Outcomes not suitable for pooling (e.g., exacerbations, quality of life) underwent narrative synthesis. We conducted planned subgroup analyses (treatment duration, age) and sensitivity analyses (leave-one-out method, model switching).

A *p*-value < 0.05 was considered statistically significant. Finally, when the outcome was constituted by 10 or more studies, the publication bias was investigated by constructing funnel plots and performing Egger’s test using Jamovi^®^ software, version 2.3, with the “MAJOR” module. This decision was made because RevMan 5.4^®^ has limited functionality for the assessment of publication bias. The Certainty of the evidence was assessed by two reviewers using the GRADE (Grading of Recommendations, Assessment, Development, and Evaluation) approach, which examines five key aspects: risk of bias, inconsistency, imprecision of estimates, indirectness of evidence, and potential publication bias. All results considered in the meta-analysis were analyzed and presented in a Summary of Findings (SoF) table. Downgrading of the certainty was applied when there was sufficient evidence of significant methodological limitations in the dimensions already described. Each of these limitations could lead to a decrease of one or two levels in the certainty of the evidence, according to the severity of the findings. Based on the methodological evaluation and the consistency of the findings, the certainty of the evidence was categorized as high, moderate, low, or very low.

## 3. Results

### 3.1. Studies Identified for the Review

A total of 1069 records were identified through searches in databases and electronic registries. After the removal of 119 duplicate records, 950 articles were examined by title and abstract, from which 881 were excluded (Cohen’s Kappa: 0.84). Sixty-nine full texts were assessed for eligibility, of which eleven could not be retrieved. Finally, 58 full-text articles were analyzed, with 44 being excluded for the following reasons: not being randomized clinical trials (*n* = 10), being clinical trial protocols (*n* = 15), being conference abstracts or letters to the editor (*n* = 1), not evaluating the outcomes of interest (*n* = 17), or not corresponding to the defined type of intervention (*n* = 1).

**Figure 1:**
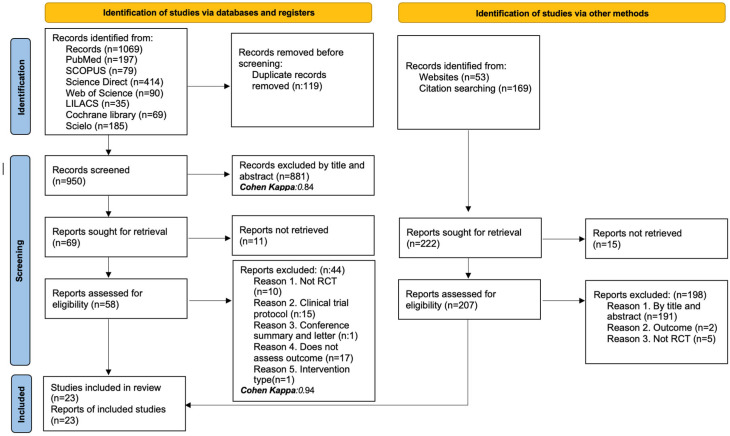
PRISMA flow diagram with the search and study selection strategy.

Additionally, 222 additional records were identified through website searches such as google scholar or consensus (*n* = 53) and citation tracking (*n* = 169). Fifteen articles were excluded for not being available, and two hundred seven were evaluated for inclusion. Of these, one hundred ninety-one studies were eliminated by title/abstract, two for not evaluating the outcomes, and the rest for not being randomized clinical trials (*n* = 5). Finally, 23 studies were included in the systematic review. The complete selection process is summarized in Figure 1.

### 3.2. Characteristics of the Studies Included in the Review

In total, twenty-three randomized clinical trials were included [37,38,39,40,41,42,43,44,45,46,47,48,49,50,51,52,53,54,55,56,57,58,59], published between 1993 and 2023, with a broad geographical representation that included countries from Europe, the Americas, Asia, and Oceania. The studies presented considerable variability in sample size, with populations ranging from 15 to 117 participants, and with relatively balanced intervention and control groups in most cases. The age range of the patients varied from 9 to 32 years, with a predominance of children and adolescents, and with a proportion of males ranging from 34% to 62%.

The duration of the programs was heterogeneous. Some studies employed brief interventions, such as that of Flores J et al. [37], which used a program of only two weeks, or that of Gungor S et al. [40], with a six-week duration. In contrast, Schneider-man J et al. [58] developed a program with a follow-up of up to 156 weeks, which is an exception among the reviewed studies. The majority of the trials used interventions lasting between 6 and 12 weeks, which can be considered the operational standard in this population.

Regarding the outcomes evaluated, pulmonary function was the most frequently reported, present in nearly twenty studies. Exercise capacity and health-related quality of life were also consistently investigated. However, only a few studies, such as that by Hebestreit H et al. [38], incorporated other clinically relevant outcomes like exacerbations and hospitalizations, in addition to adverse effects. This trial was also one of the most methodologically robust, with a large sample size and an international multicenter design, which reinforces the external validity of its findings.

Regarding provenance, a significant contribution from studies conducted in Brazil, the United Kingdom, Turkey, and Spain is observed, which could be related to the local infrastructure for cystic fibrosis care and academic interest in rehabilitation strategies. Furthermore, some authors, such as Santana et al. [50,51], appear in more than one publication, suggesting continuity in their lines of research (see Table 1).

### 3.3. Characteristics of the Population and the Applied Intervention

The included studies show differences regarding the implementation context of the interventions in patients with cystic fibrosis. Concerning the setting of care, it was identified that the majority of interventions were implemented in a hospital setting, accounting for 61% of the total included studies [37,38,39,40,46,47,51,52,53,54,56,57,58,59]. This predominance of the institutional environment could be associated with the availability of infrastructure, specialized equipment, and direct supervision by health professionals. On the other hand, 30% of the studies conducted the interventions in the participants’ homes, which represents an alternative with the potential for greater adherence and sustainability, especially in contexts where frequent outpatient visits are not feasible [41,42,44,45,48,49,55]. Two studies (9%) used a mixed model, which combined home-based and institutional sessions, demonstrating the exploration of more flexible care schemes in this population [43,50].

Among the home-based studies, the works of Del Corral T et al. [45] and Gupta S et al. [42] stand out. The former applied an intervention based on active video games, while the latter used resistance training, both designed to be performed without continuous in-person supervision. These strategies highlight the potential of accessible technologies and the adaptability of exercise programs to non-institutional settings. In contrast, studies like those by Schneiderman J et al. [58], and Jong W et al. [57] were conducted in clinical settings, which allowed for the implementation of modalities such as respiratory physiotherapy, inspiratory muscle training, and structured aerobic exercise with strict professional control.

The activities integrated into the interventions were heterogeneous in their nature, intensity, and complexity. Aerobic training (walking, stationary cycling, treadmill), respiratory physiotherapy, inspiratory muscle training, electrostimulation, and active video games were frequently reported. Some studies combined more than one strategy, reflecting a multimodal approach. For example, Schneiderman J et al. [58] executed their intervention over 468 sessions, being the protocol with the highest total intervention load, while Gungor S et al. [40] implemented a significantly smaller number of six sessions, focused on muscle training. This spectrum of intensity and duration highlights the lack of standardization in physical rehabilitation programs aimed at people with cystic fibrosis (see Table 2).

### 3.4. Results of the Risk of Bias Assessment

**Figure 2:**
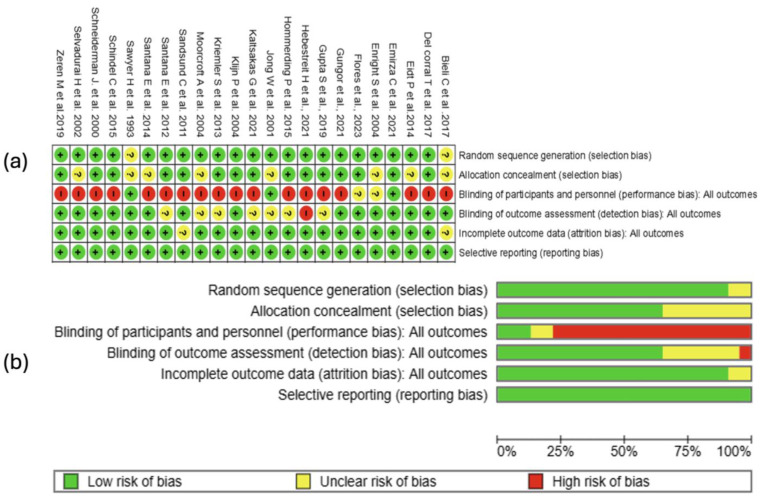
Risk of bias evaluation for the studies included in this review. (**a**) The “+” symbol denotes low risk of bias, “?” indicates unclear risk, and “−” represents high risk of bias. The colors associated with each symbol are green for low risk, yellow for unclear risk, and red for high risk. (**b**) This panel shows a summary of the identified risk of bias across all evaluated studies, displaying the percentage corresponding to each risk-of-bias item [37,38,39,40,41,42,43,44,45,46,47,48,49,50,51,52,53,54,55,56,57,58,59].

The risk of bias analysis of the studies included in the review was conducted considering various domains, as illustrated in Figure 2. The assessment was carried out using the RevMan 5.4^®^ tool (accessed on 30 April 2025), which facilitated the identification of robust aspects and dimensions susceptible to improvement in the design and execution of the evaluated clinical trials.

#### 3.4.1. Random Sequence Generation

The domain corresponding to random sequence generation was rated as low risk of bias in 21 studies [37,38,39,40,41,42,43,45,46,47,48,49,50,51,52,53,54,55,56,57,58]. This high proportion suggests an adequate and clearly described application of randomization methods, which is an essential component for preserving the internal validity of the trials. However, in two studies [44,59], an unclear risk was assigned due to insufficient methodological information to judge with certainty the rigor of the randomization employed. The low prevalence of bias risk in this domain reinforces the methodological soundness of most of the included studies and supports the reliability of the effects estimated in this meta-analysis.

#### 3.4.2. Allocation Concealment

Regarding the allocation concealment domain, 15 of the 23 included randomized clinical trials were rated as low risk of bias [37,38,39,40,41,42,43,45,46,47,49,50,51,53,58], indicating an adequate implementation of strategies to prevent the prediction of the assigned group before participant inclusion. However, in eight studies [44,48,50,54,55,56,57,59] an unclear risk was assigned due to the lack of sufficient information about the mechanisms used to ensure concealment, such as the use of opaque, sealed envelopes or centralized randomization systems. This relatively high proportion of methodological uncertainty in this domain could introduce a potential selection bias.

#### 3.4.3. Blinding of Participants and Personnel

Regarding the domain of blinding of participants and personnel, eighteen of the twenty-three included studies were rated as high risk of bias, while two presented unclear risk [37,54], and only three studies managed to implement adequate strategies to minimize this type of bias [41,57,59]. This trend is understandable, considering the nature of the evaluated interventions (mainly rehabilitation programs in patients with cystic fibrosis), where complete blinding is often difficult or even infeasible for practical and ethical reasons. Nevertheless, the high risk of bias in this domain could have influenced the participants’ subjective perception of the treatment effects, as well as the behavior of the personnel responsible for the intervention, which should be taken into account when interpreting the magnitude of the observed effects.

#### 3.4.4. Blinding of Outcome Assessment

Regarding the domain related to the blinding of outcome assessors, the majority of the studies [37,40,41,43,44,45,46,48,51,52,53,54,56,58,59] were classified as low risk of bias, indicating that in most of the included trials, appropriate measures were taken to minimize the influence of assessors on the measurement of results. However, seven studies [39,42,47,49,51,55,57] presented unclear risk due to the lack of clear information on the implementation of masking procedures, and one study [38] was evaluated as high risk, suggesting a possible susceptibility to observation bias. Although most studies showed adequate protection against this type of bias, the non-negligible proportion of methodological uncertainty in this domain highlights the need to improve transparency in the description of outcome assessment methods in future trials.

#### 3.4.5. Incomplete Outcome Data

In the domain related to incomplete outcome data, 21 of the 23 randomized clinical trials [37,38,39,40,41,42,43,45,46,47,48,49,50,51,53,54,55,56,57,58,59] were classified as low risk of bias, indicating adequate management of losses to follow-up and the use of appropriate analyses in the majority of the studies. Only two studies (8.7%) were evaluated as having an unclear risk due to the absence of sufficient information to judge the potential impact of participant losses or exclusions on the results [44,52]. The high prevalence of low risk in this domain suggests that the effects estimated in the meta-analysis were not substantially affected by biases related to the handling of incomplete data.

#### 3.4.6. Selective Reporting

Regarding bias due to selective reporting of outcomes, all included studies were rated as low risk. This consistency suggests that the clinical trials reported all pre-specified outcomes in their protocols or registries completely and transparently, without evidence of systematic omission of unfavorable or non-significant outcomes (see Figure 2a).

#### 3.4.7. Summary of Risk of Bias

The risk of bias assessment revealed that, in general, the included clinical trials presented adequate methodological quality in most of the analyzed domains. The generation of the random sequence and the handling of incomplete data were adequate in more than 90% of the studies. Allocation concealment showed some degree of uncertainty (35% with unclear risk). Blinding of participants and personnel was the most compromised domain, with a high risk in 78% of the studies, likely due to the nature of the intervention. Regarding the blinding of assessors, most studies presented a low risk, although there was a non-negligible percentage of studies with unclear or high risk. All studies reported their outcomes completely and without selective bias. Taken together, these findings support the internal validity of the results, although they highlight the need to improve blinding procedures in future research (see Figure 2b).

### 3.5. Qualitative Synthesis of the Scientific Evidence

#### 3.5.1. Exacerbations

One of the twenty-three studies analyzed pulmonary exacerbations as an outcome of interest. In this regard, Hebestreit H et al. [38], in a population of 117 patients homogeneously distributed into vigorous physical training and standard care groups, found that subjects in the intervention arm presented a higher number of exacerbations than those assigned to the control group (45% versus 40%); however, the difference was not statistically significant.

#### 3.5.2. Hospitalization

In the RCT conducted by Hebestreit et al. [38], the effect of a structured, individualized physical exercise program on the rate of hospitalizations was examined in a cohort of 117 patients with cystic fibrosis. Participants were randomly assigned to an intervention group, which received supervised training, or a control group with standard care. After a 12-month follow-up period, the proportion of hospitalized patients showed no statistically significant differences between the groups (37.9% in the intervention group versus 34.5% in the control group).

#### 3.5.3. Health-Related Quality of Life

Of the 23 randomized clinical trials included in this review, 13 studies (56.5%) evaluated quality of life as an outcome, using different measurement instruments. The most frequently used questionnaires were the Cystic Fibrosis Questionnaire (CFQ) in five studies [41,44,47,52,53] (38.5%) and its revised version, the CFQ-R, in 30.8% of the studies [37,39,40,45]. Other instruments used were the Chronic Respiratory Disease Questionnaire (CRDQ) [54] in one study, as was the SF-36 Health Survey [48]. In two studies (15.4%), the authors did not specify the instrument used.

Regarding the findings, eight of the thirteen studies did not report statistically significant differences between the intervention group and the control group in the evaluated domains of quality of life, suggesting limited evidence on the impact of the interventions on this outcome. In contrast to the previously described findings, studies such as those by Emirza C. et al. [41], Flores J et al. [37], Gungor S et al. [40], Klijin P et al. [53], and Del Corral et al. [45] reported significant improvements in quality of life in certain domains, such as vitality, emotional state, and physical functioning, in participants assigned to pulmonary rehabilitation or physical training programs.

#### 3.5.4. Adverse Events

Of the 23 studies included in the review, 34.8% [38,41,43,49,50,51,52,53] reported specific information on adverse events related to the interventions. In most of these trials, no adverse events directly attributable to the pulmonary rehabilitation or physical training programs were documented. When side effects were reported, such as isolated cases of dehydration or fatigue, they were classified as mild and not causally related to the intervention. Some studies reported the occurrence of serious adverse events, although their frequency was low, and no statistically significant differences were observed between the intervention and control groups [38]. These findings, although limited by the variability in the methods of recording adverse events, support the general safety of non-pharmacological interventions in patients with cystic fibrosis.

### 3.6. Meta-Analysis

A meta-analysis was performed for the outcomes of pulmonary function (FEV1, FVC, FEV1/FVC, and RV/TLC) and exercise capacity (VO_2_ max, 6MWT, and Wmax), based on the studies by Flores J et al. [37], Hebestrit H et al. [38], Kaltsakas G et al. [39], Gungor et al. [40], Emirza C et al. [41], Gupta S et al. [42], Zeren M et al. [43], Bieli C et al. [44], Del corral T et al. [45], Schindel C et al. [46], Hommerding P et al. [47], Eidt P et al. [48], Kriemler S et al. [49], Santana et al. [50,51], Sandsund C et al. [52], klijn P et al. [53], Enright S et al. [54], Moorcroft A et al. [55], Selvadurai et al. [56], Jong W et al. [57], Schneiderman J et al. [58], and Sawyer H et al. [59].

#### 3.6.1. Results of the Evidence Quality Assessment

Of the twenty-three studies evaluated, four studies (17.4%) obtained the maximum score of 5 points, indicating complete adherence to the assessed domains [37,41,54,57]. The majority of the studies (78.3%) obtained a score of 4 points, mainly due to the absence of double-blinding, a frequent limitation in research with non-pharmacological interventions such as physical exercise or pulmonary rehabilitation, where masking the participant and personnel is often not feasible. Only one study [52] obtained a score of 3, due to the lack of reporting on the management of withdrawals during follow-up. Despite these differences, all included studies were randomized, used adequate allocation procedures, and clearly defined their inclusion and exclusion criteria (see details in Table 3).

#### 3.6.2. Pulmonary Function

##### Forced Expiratory Volume in the First Second (FEV1)

**Figure 3:**
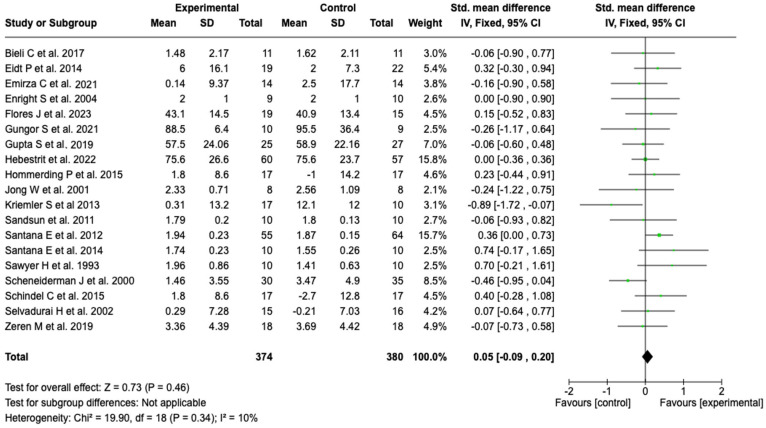
Forest plots of the effect of pulmonary rehabilitation and physical training on FEV1 in patients with cystic fibrosis [37,38,40,41,42,43,44,46,47,48,49,50,51,52,54,56,57,58,59]. No clinically significant effect was observed; low heterogeneity (I^2^ = 10%) supports consistency across studies.

Nineteen studies (*n* = 754) evaluated the effect of the intervention on FEV_1_. The meta-analysis showed no statistically significant difference between the groups (SMD: 0.05; 95% CI: −0.09 to 0.20; *p* = 0.46). Heterogeneity was low (I^2^ = 10%), suggesting consistency among the studies. From a clinical standpoint, these findings indicate that pulmonary rehabilitation or physical training does not have a relevant effect on FEV_1_ (see Figure 3). No subgroup analyses were performed due to low heterogeneity. Sensitivity analyses conducted by modifying the statistical model and by sequential exclusion of individual studies did not substantially change the direction or magnitude of the effect, which reinforces the robustness of the results (see Figures S1–S4 of the Supplementary Material).

##### Forced Vital Capacity (FVC)

**Figure 4:**
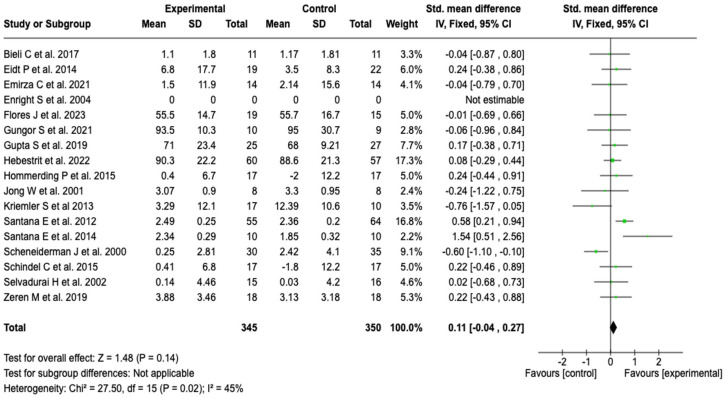
Forest plots of the effect of pulmonary rehabilitation and physical training on FVC in patients with cystic fibrosis [37,38,40,41,42,43,44,46,47,48,49,50,51,54,56,57,58]. No statistically significant differences were observed between the experimental and control groups. Moderate heterogeneity was found (I^2^ = 45%), which may limit the precision of the estimates.

Seventeen studies (*n* = 695) were analyzed to evaluate the effect on FVC. No significant differences were observed between groups (SMD: 0.11; 95% CI: −0.04 to 0.27; *p* = 0.14), with a heterogeneity of 45% (see Figure 4). The robustness of the findings was confirmed through sensitivity analyses, by applying alternative meta-analysis models and by individually excluding the included studies; in no case was the direction or magnitude of the effect modified (see Figures S5–S8 of the Supplementary Material).

##### FEV1/FVC Ratio

**Figure 5:**
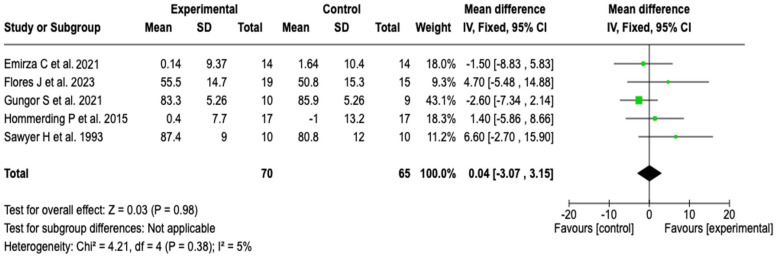
Forest plots of the effect of pulmonary rehabilitation and physical training on the FEV1/FVC ratio in patients with cystic fibrosis [37,40,41,47,59]. No significant differences were observed between groups (*p* = 0.98; I^2^ = 5%). The studies showed low heterogeneity and consistent results.

Five studies (*n* = 135) evaluated the FEV1/FVC ratio. The meta-analysis showed no significant differences between the groups (MD: 0.04; 95% CI: −3.07 to 3.15; *p* = 0.98), with low heterogeneity (I^2^ = 5%). From a clinical perspective, this result indicates that pulmonary rehabilitation or physical training did not have a relevant effect on the FEV1/FVC ratio (see Figure 5). The sensitivity analysis, performed by switching to a random-effects model, did not alter the direction or magnitude of the effect, which reinforces the robustness of the finding (see Figure S9 of the Supplementary Material).

##### Residual Volume to Total Lung Capacity Ratio (RV/TLC)

**Figure 6:**
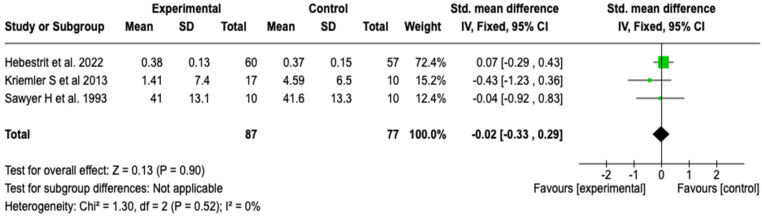
Forest plots of the effect of pulmonary rehabilitation and physical training on the RV/TLC ratio in patients with cystic fibrosis [38,49,59]. No differences were found between groups. The results were consistent across studies with no heterogeneity observed (I^2^= 0%).

Three studies with a total of 164 patients evaluated the effect of the intervention on the RV/TLC ratio. The meta-analysis did not show a statistically significant difference between the groups (SMD: −0.02; 95% CI: −0.33 to 0.29; *p* = 0.90), with null heterogeneity (I^2^ = 0%). These results suggest that the intervention did not have a clinically relevant impact on air trapping or pulmonary hyperinflation (see Figure 6). A sensitivity analysis was performed using the random-effects model, with no relevant changes in the direction or magnitude of the estimated effect (see Figure S10 of the Supplementary Material).

#### 3.6.3. Exercise Capacity

##### 6-Minute Walk Distance (6MWD)

**Figure 7:**
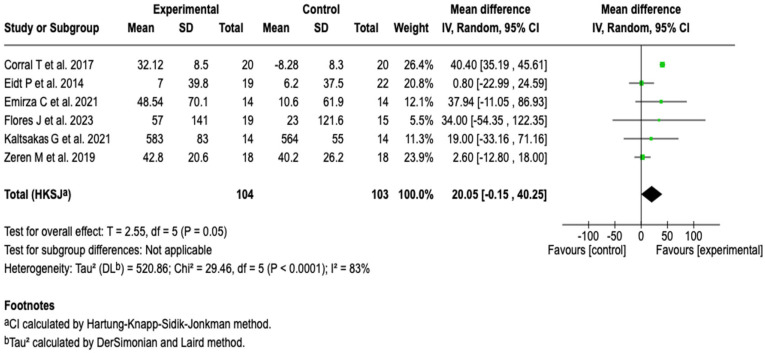
Forest plots of the effect of pulmonary rehabilitation and physical training on the 6-minute walk distance in patients with cystic fibrosis [37,39,41,43,45,48].

Six studies (*n* = 207) analyzed the impact of the intervention on the distance covered in the six-minute walk test. The meta-analysis conducted under a random-effects model showed a mean difference of 20.05 m in favor of the experimental group; however, this was not statistically significant (95% CI: −0.15 to 40.25; *p* = 0.05) (see Figure 7). Heterogeneity among studies was high (I^2^ = 83%), so subgroup analyses were performed according to age group and treatment duration, without identifying important differences (see Figures S11 and S12 of the Supplementary Material). Likewise, the sensitivity analysis using an alternative model did not substantially modify the magnitude or direction of the effect (see Figure S13 of the Supplementary Material).

##### Oxygen Consumption (VO_2_ Max)

**Figure 8:**
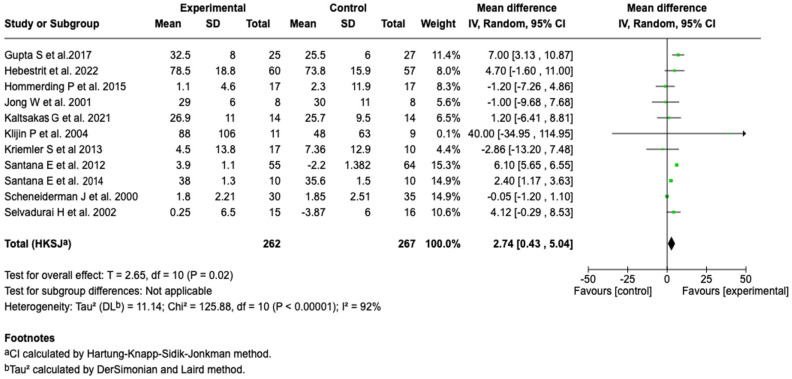
Forest plots of the effect of pulmonary rehabilitation and physical training on VO_2_ max in patients with cystic fibrosis [38,39,42,47,49,50,51,53,56,57,58]. The experimental group showed a significant increase in VO_2_. High heterogeneity (I^2^ = 92%) was observed.

Eleven studies (*n* = 529) evaluated the effect of the intervention on oxygen consumption. The meta-analysis showed a significant mean difference in favor of the pulmonary rehabilitation or physical training group (MD: 2.74; 95% CI: 0.43 to 5.04; *p* = 0.02), which indicates an improvement in VO_2_ max with the intervention. However, heterogeneity was considerable (I^2^ = 92%), suggesting high variability among the included studies and limiting the precision of the overall estimate (see Figure 8).

The analysis by age subgroups showed a significant effect in the pediatric population (MD: 3.27; 95% CI: 0.03 to 6.50; *p* = 0.05) but not in adults (MD: 1.52; 95% CI: −3.67 to 6.72; *p* = 0.42). However, no statistically significant difference was evidenced between age subgroups (*p* = 0.49). Heterogeneity was high in pediatrics (I^2^ = 95%) and null in adults (I^2^ = 0%) (see Figure S14). On the other hand, the subgroup analysis according to treatment duration also showed no statistically significant differences (*p* = 0.73) (see Figure S15 of the Supplementary Material).

Additionally, the sensitivity analysis based on the individual removal of the study by Kriemler S et al. [49] maintained the direction and increased the size of the effect in favor of the intervention group, with persistence of statistical significance (MD: 3.0; 95% CI: 0.65 to 5.34; *p* = 0.02) (see Figure S16). This behavior was not maintained when removing the study by Selvadurai H et al. [56] (MD: 2.56; 95% CI: −0.03 to 5.15; *p* = 0.05) (see Figure S17).

##### Wmax

**Figure 9:**
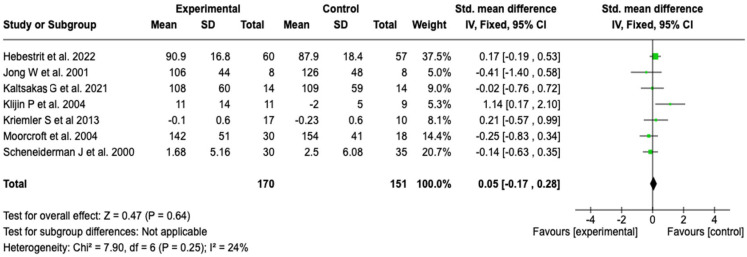
Forest plots of the effect of pulmonary rehabilitation and physical training on Wmax in patients with cystic fibrosis [38,39,49,53,55,57,58]. No significant difference in Wmax was found between the experimental and control groups (I^2^ = 24%).

The effect of pulmonary rehabilitation on Wmax was evaluated in seven studies that included a total of 321 patients (170 in the experimental group and 151 in the control group). The results of the meta-analysis showed a standardized mean difference of 0.05 (95% CI: −0.17 to 0.28; *p* = 0.64), which indicates that pulmonary rehabilitation or physical training did not have a statistically significant effect on Wmax compared to usual care (see Figure 9). Heterogeneity among the studies was low (I^2^ = 24%), so it was not investigated through subgroup analysis. Furthermore, the sensitivity analysis, performed both by meta-analysis model and by sequential removal of individual studies, did not modify either the direction or the size of the effect, which reinforces the robustness and stability of the findings (see Figures S18 and S19 of the Supplementary Material).

In Table S1 of the Supplementary Material, the main findings of the meta-analysis are summarized, including the number of studies, effect size with its 95% confidence interval, and the certainty of the evidence.

#### 3.6.4. Publication Bias

**Figure 10:**
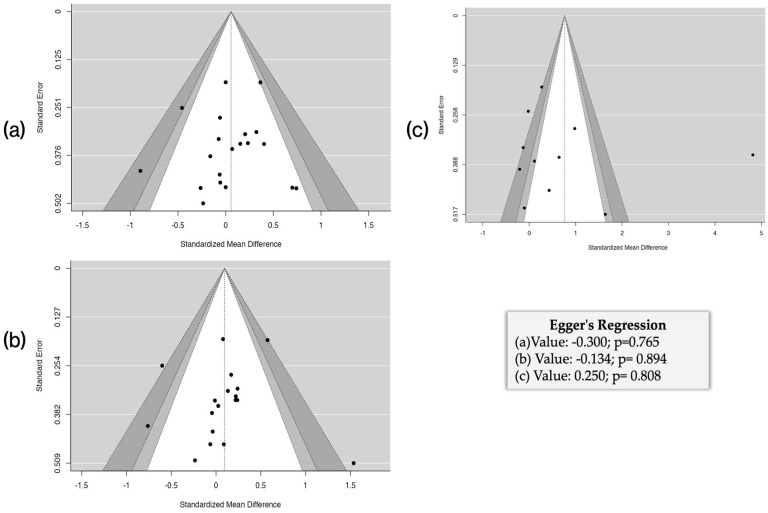
Evaluation of publication bias using funnel plots and Egger’s regression test for the outcomes FEV_1_ (**a**), FVC (**b**), and VO_2_ (**c**).

The risk of publication bias was assessed using funnel plots for the outcomes: FEV1 (Figure 10a), FVC (Figure 10b), and VO_2_ max (Figure 10c). Visually, the plots showed a relatively symmetrical distribution of the studies around the standardized mean effect, with no clear evidence of substantial asymmetry. This suggests a low probability of visually detectable publication bias. To complement the visual analysis, Egger’s regression test was applied, which did not yield statistically significant results in any of the analyzed outcomes. This result reinforces that there were no publication biases in the evaluated outcomes.

#### 3.6.5. Results of the GRADE Certainty of Evidence Assessment

The certainty of the evidence was low for most of the outcomes related to pulmonary function (FEV1, FVC, FEV1/FVC, RV/TLC) and exercise capacity (VO_2_ max and Wmax), particularly due to problems related to imprecision. The 6MWT was rated as having very low certainty, derived from high inconsistency, imprecision, and high risk of bias in the studies that support it. These limitations reduce confidence in the estimated effects and suggest that future research could substantially modify these results (see details in Table 4 and Table S2 of the Supplementary Material).

## 4. Discussion

### 4.1. Main Findings of the Review

The objective of this meta-analysis was to determine the effectiveness of pulmonary rehabilitation and physical training in patients with cystic fibrosis. A total of 23 randomized controlled trials were included, involving more than 800 patients evaluated in various clinical settings. The findings show that the evaluated interventions did not produce significant effects on parameters related to pulmonary function (FEV1, FVC, FEV1/FVC, RV/TLC). These results, supported by low heterogeneity and consistent sensitivity analyses, suggest that pulmonary rehabilitation and physical training do not substantially modify ventilatory mechanics in patients with cystic fibrosis.

In contrast to the above, a significant improvement was observed in maximal oxygen uptake, with a mean difference of 2.74 mL/kg/min (95% CI: 0.43 to 5.04; *p* = 0.02), which suggests greater efficiency of the cardiorespiratory system during exercise. A favorable trend was also identified in the six-minute walk distance (MD: 20.05 m; 95% CI: −0.15 to 40.25; *p* = 0.05), although it did not reach statistical significance and was accompanied by high heterogeneity. These results point to a possible functional benefit of the interventions, focused on improving exercise capacity rather than on reversing structural or spirometric alterations.

In addition to the quantitative findings, the qualitative synthesis provides complementary elements that offer a broad overview of the clinical utility of the analyzed interventions. In terms of pulmonary exacerbations and hospitalizations, only one study evaluated these outcomes, without demonstrating statistically significant differences between the intervention and control groups. On the other hand, regarding health-related quality of life, although no significant benefits were observed in most studies, some works reported specific improvements in particular domains such as vitality, emotional state, or physical functioning. This suggests possible positive effects that are limited and not consistent across all evaluated dimensions. Regarding safety, adverse events were described in one-third of the studies and were generally mild manifestations, such as fatigue or dehydration. No significant differences were identified in the frequency of serious events between groups, which reinforces the safety profile of these non-pharmacological interventions.

### 4.2. Comparison with Previous Studies

The assessment of the evidence was carried out using the Jadad scale, which allowed for an adequate discrimination of the quality of the studies related to aspects such as randomization, blinding, and selection criteria [60]. Additionally, robust statistical strategies were applied to quantify heterogeneity between studies, as well as to explore possible sources of variability through sensitivity and subgroup analyses when the quantity and consistency of the data permitted. The overall certainty of the evidence for each outcome was rated following the guidelines of the GRADE approach, which allowed for a more precise establishment of the robustness and clinical applicability of the obtained results [61]. This methodological approach, based on standard validated tools, gives the present analysis greater consistency and interpretive depth compared to previous reviews on the subject.

A large portion of previously conducted reviews agree that the effect of pulmonary rehabilitation and physical training on pulmonary function and exercise capacity in patients with CF is questionable. Despite this, most express serious limitations in the included studies, which in most cases prevents making open recommendations.

In this regard, a meta-analysis by García-Pérez et al. [34], which included a total of 12 studies on physical training in patients with CF, found that the interventions produced limited benefits on pulmonary function measured by spirometry. However, they more consistently improved aerobic capacity and certain muscle strength parameters, especially in adults. It is necessary to highlight that the authors warned that a significant limitation of the analyzed studies was the small sample size in many of them, which could have influenced the statistical power of the results.

Similarly, in an analysis that included six studies with a total of 151 participants, the pooled data showed no significant differences in pulmonary function or exercise capacity between the treatment and control groups. However, subgroup analyses suggested that inspiratory muscle training could be beneficial for increasing maximal inspiratory pressure, although this outcome was not considered in this investigation [62]. In the same vein, Stanford G et al. [63] reported that respiratory muscle training in patients with CF does not produce significant differences between groups in terms of pulmonary function (FEV1 and FVC) or exercise capacity measured by VO_2_ max or the six-minute walk test.

In contrast to the previous reports, a Cochrane review by Radtke T et al. [64] with 487 participants observed that aerobic or anaerobic physical training (or a combination of both) has positive effects on aerobic exercise capacity, pulmonary function, and health-related quality of life, which is consistent with other past reviews [65]. Despite this, among the limitations, the authors highlight low methodological quality, heterogeneity in the training programs, and inconsistency in the results, which makes it difficult to draw definitive conclusions.

It is important to mention that the results of our study regarding FEV_1_ are consistent with previous research that has questioned the impact of regular physical exercise on FEV_1_ in people with cystic fibrosis [66]. The evidence suggests that it is unlikely that relevant improvements in FEV_1_ will occur solely through exercise, because this measure primarily reflects airway obstruction and is usually influenced by chronic structural alterations of the pulmonary parenchyma, which are not easily modifiable with non-pharmacological interventions alone [67]. Additionally, it should be considered that FEV_1_, although a spirometric parameter frequently used as a prognostic marker in cystic fibrosis, might not be the most sensitive measure for detecting the functional benefits of exercise, especially in the short term [64].

The consistency between our findings and those of previous reviews [34,62,63], regarding the limited impact of exercise on pulmonary function and some markers of exercise capacity in patients with cystic fibrosis, can be explained by the irreversible structural alterations of the lung parenchyma, chronic bronchial remodeling, and persistent airway obstruction, which limit the functional response to physical interventions [68,69]. Furthermore, secondary muscle dysfunction due to deconditioning and the systemic inflammatory burden can attenuate the expected benefits of training, especially when the progression of the disease is not controlled [70].

In our review, patients who received pulmonary rehabilitation or physical training had a higher maximal VO_2_ level. This finding is important, given that maximal VO_2_ has been described as a crucial prognostic marker in patients with CF, due to its well-established link with mortality risk and the clinical progression of the disease [71]. Although the available evidence has been limited, some studies included in systematic reviews have reported significant increases in maximal VO_2_, particularly after interventions focused on inspiratory muscle training, which supports the physiological plausibility of our results [64]. These effects suggest that, under certain conditions of intensity, duration, and adherence, physical training could induce sufficient cardiovascular and muscular adaptations to improve maximal oxygen consumption, even in the presence of the pulmonary limitations characteristic of CF [72,73].

Despite the positive findings in terms of VO_2_, it is important to mention the high heterogeneity observed among the included studies (I^2^ = 92%). This variability can be attributed to multiple factors related to the clinical and methodological characteristics of the research. For example, the type of intervention varied considerably: some studies used conventional aerobic programs [38,58], while others included inspiratory muscle training [50,57] or combined approaches, which can lead to substantial differences in the results. Likewise, the study population was not homogeneous, including both pediatric and adult patients, who might respond differently to exercise due to factors such as the degree of pulmonary involvement or the capacity for treatment adherence. The setting in which the interventions were implemented must also be considered: studies with supervised programs in a hospital setting [38,39] may obtain more consistent effects compared to those conducted at home [41,42,45].

Although some comparisons with previous studies have been made in this discussion, it is important to highlight that the available evidence on physical training and pulmonary rehabilitation in patients with cystic fibrosis remains limited. There are few systematic reviews or meta-analyses focused on physical interventions in this population, which makes it difficult to establish broad and solid comparisons. This scarcity of previous evidence syntheses represents a limitation for fully contextualizing the findings of the present study.

### 4.3. Limitations of the Included Studies

Several limitations that require discussion were evident in the included studies. First, most of the included studies had small sample sizes, generally fewer than 100 participants, which compromises the statistical power of the analyses and limits the ability to detect clinically significant effects. This limitation increases the probability of type II errors and reduces the reliability of the effect estimates, thus hindering the precise interpretation of the impact of rehabilitation interventions in patients with cystic fibrosis [74].

On the other hand, there is marked heterogeneity in the duration of interventions and follow-up periods among the included studies, ranging from 2 to 156 weeks [37,58]. This variability limits the ability to establish solid conclusions about the sustainability of the observed effects. Although benefits in functional capacity were evidenced in the short term, evidence on their long-term maintenance remains scarce and inconsistent. In addition to this, the studies show considerable differences in the type of exercise used, frequency, duration of each session, intensity, and setting, as well as in the outcome measures used. This variability makes direct comparison between studies difficult and can influence the magnitude of the observed effect.

A recurring methodological limitation in the included studies was the high risk of blinding bias, for both participants and assessors. This weakness is expected in interventions such as pulmonary rehabilitation and physical training, where it is inherently difficult to mask group allocation due to the active and evident nature of the interventions [75].

Finally, the examined studies were conducted primarily in high-income countries like the United Kingdom, which raises questions about the validity of the results in low and middle-income contexts. Factors such as access to health services, availability of technology, and socioeconomic conditions can significantly impact the results [76]. Furthermore, the study population consisted mostly of adolescents and young adults.

Taken together, these methodological and contextual limitations not only compromise the robustness of the available evidence but also highlight the need for better-designed future studies, with more representative samples, standardized interventions, and greater rigor in bias control. Furthermore, it is crucial that more diverse populations be included and that low- and middle-income contexts be considered, where the implementation of these strategies may face additional challenges.

### 4.4. Limitations of the Review

The meta-analysis showed moderate to high levels of statistical heterogeneity in some outcomes, illustrating the variability in study designs, applied interventions, and participant characteristics. This reduces the certainty of the overall effect estimates and suggests that the benefits of physical training may depend on the specific context of each intervention. On the other hand, an aspect to highlight is that, due to omission in the studies, a broad analysis of key outcomes such as hospitalization rates and the frequency of pulmonary exacerbations could not be performed, despite their pathophysiological relevance in the progression of CF. This omission may be because these events require prolonged observation periods and robust clinical registration systems, which is not always feasible in studies with short-term designs. Furthermore, the variability in the operational definition of a CF exacerbation and the influence of multiple factors such as infections, treatment adherence, and environment make their use as primary outcomes difficult.

An important methodological aspect is that most of the included studies did not conduct follow-up after the intervention program, which prevents the evaluation of the sustainability of the observed benefits in exercise capacity. This gap limits the understanding of the long-term impact of pulmonary rehabilitation and physical training in CF. Additionally, although our review chose to integrate data from pediatric and adult populations to broaden the scope of the analysis, this decision introduces relevant clinical heterogeneity. The differences in pulmonary pathophysiology, the degree of systemic involvement, the response to exercise, and treatment adherence among children, adolescents, and adults with CF can significantly modify the outcomes of the interventions, making a unified interpretation of the results difficult.

A possible limitation of the present study is the exclusion of preprints, defined a priori to ensure the exclusive inclusion of peer-reviewed evidence. This methodological decision, while strengthening the quality of the analyzed body of evidence, may restrict the incorporation and analysis of recent studies not yet formally published. However, after a thorough review, no relevant preprints were identified that could have substantially modified the results or conclusions of this meta-analysis.

The GRADE analysis showed that most of the evaluated outcomes had low or very low certainty of evidence. This rating reflects recurring methodological limitations in the included studies, such as risk of bias, small sample sizes, imprecision in the results, and the heterogeneity presented in the meta-analysis of some outcomes. These deficiencies restrict confidence in the findings and hinder the formulation of firm clinical recommendations on the effect of rehabilitation and physical training in patients with cystic fibrosis. Therefore, it is imperative to have future research that employs more rigorous designs, greater statistical power, and standardization in the evaluated outcomes to consolidate the evidence in this field.

### 4.5. Strengths of the Review

This meta-analysis makes a significant contribution by updating the available evidence with more recently published randomized controlled trials, which allows for a more faithful reflection of the current state of knowledge on pulmonary rehabilitation and exercise in patients with cystic fibrosis. Furthermore, the incorporation of a rigorous methodological approach through the combined use of the Jadad scale, the RoB 2 tool for assessing the risk of bias, and the GRADE system for rating the certainty of the evidence, strengthens the internal validity and clinical applicability of the findings. This comprehensive evaluation strategy not only improves the quality of the analysis but also allowed for a more precise identification of the strengths and limitations of the synthesized evidence.

Likewise, this systematic review is distinguished by having included randomized controlled trials published over a wide time range, from 1990 to 2024. This methodological decision allowed for the integration of a representative and heterogeneous sample of studies, increasing the extrapolation of the findings without restricting the analysis to a specific period. The inclusion of studies published over more than three decades provides a more comprehensive view of the body of evidence available on physical training and pulmonary rehabilitation in patients with cystic fibrosis.

### 4.6. Clinical Implications

The findings of this review and meta-analysis show that, although rehabilitation and exercise interventions in patients with cystic fibrosis do not produce significant improvements in pulmonary function, quality of life, frequency of exacerbations, or hospitalizations, they do generate a positive impact on aerobic capacity, especially in maximal oxygen consumption. This effect is clinically relevant, given that exercise capacity is closely related to survival and prognosis in this population [71]. Therefore, rehabilitation should prioritize activities that promote improvements in aerobic capacity, favoring cardiovascular and muscular adaptations that contribute to optimizing the patient’s functional status. However, given the absence of changes in other critical outcomes, it is necessary to consider rehabilitation as a complement within a comprehensive and multidisciplinary approach, without expecting it to modify pulmonary progression or disease-associated clinical events on its own. Finally, the implementation of individualized programs and the promotion of long-term exercise adherence are fundamental aspects to maximize the observed functional benefits.

### 4.7. Future Recommendations

Future research should focus on conducting randomized clinical trials with larger sample sizes and prolonged follow-up periods to more accurately evaluate the effects of rehabilitation and physical training in patients with cystic fibrosis. Likewise, it is essential to standardize outcome variables, such as forced expiratory volume in the first second, forced vital capacity, the six-minute walk test, cystic fibrosis-specific quality of life questionnaires, and maximal oxygen consumption, to facilitate comparison between studies and allow for the development of more solid clinical guidelines. It is recommended to explore the use of remote technologies and home-based strategies, such as mobile applications, wearable devices, and telemedicine, which can improve adherence and make the intervention more accessible and sustainable.

Technology-enhanced home-based interventions, such as the use of active video games or remote supervision, represent a promising avenue for expanding access to rehabilitation programs. These strategies not only promote the continuity of treatment at home but also allow for greater personalization and monitoring of the patient’s progress. In an environment where telehealth and patient-centered care are gaining prominence, these types of approaches can improve adherence, reduce logistical barriers, and facilitate larger-scale implementation, especially in resource-limited contexts. Their progressive integration into hybrid care models could strengthen the effectiveness and sustainability of home-based interventions. The active role of respiratory therapists and interdisciplinary teams will be crucial for the effective implementation of these evidence-based approaches.

## 5. Conclusions

This systematic review and meta-analysis demonstrate that, in patients with cystic fibrosis, interventions based on pulmonary rehabilitation and physical exercise do not show a consistent effect on clinically critical outcomes such as pulmonary function, quality of life, exacerbations, or hospitalizations. However, a significant improvement is identified in exercise capacity, particularly in maximal oxygen consumption, a functional parameter with strong prognostic value in this population. These findings underscore the importance of integrating exercise as an essential component of therapeutic strategies, not as an isolated measure, but within the framework of a multidimensional approach. Due to the low certainty of the evidence and the considerable methodological heterogeneity among the included studies, the results of this review should be interpreted with caution in clinical practice.

## Figures and Tables

**Table 1:** Characteristics of the studies included in the review.

Author, Year	Country	Patients	Sex (% Male)	Age (Years)	Program Duration (Weeks)	Outcomes Assessed
Flores J et al. [37], 2023	Brazil	*n*: 34, I: 19, C: 15	62%	28	2	Pulmonary function, exercise capacity, health-related quality of life.
Hebestrit H et al. [38], 2021	International	*n*: 117, I: 60, C: 57	44%	24	52	Pulmonary function, exercise capacity, exacerbations, hospitalizations, health-related quality of life, and adverse effects.
Kaltsakas G et al. [39], 2021	Greece and UK	*n*: 24, I: 12, C: 12	54%	32	12	Exercise capacity, health-related quality of life.
Gungor S et al. [40], 2022	Turkey	*n*: 19, I: 10, C: 9	58%	9	6	Pulmonary function, health-related quality of life.
Emirza C et al. [41], 2021	Turkey	*n*: 28, I: 14, C: 14	43%	13	6	Pulmonary function, exercise capacity, health-related quality of life, adverse effects.
Gupta S et al. [42], 2019	India	*n*: 52, I: 25, C: 27	58%	12.5	52	Pulmonary function, exercise capacity, health-related quality of life.
Zeren M et al. [43], 2019	Turkey	*n*: 36, I: 18, C: 18	47%	13	8	Pulmonary function, exercise capacity, and adverse events.
Bieli C et al. [44], 2017	Switzerland	*n*: 22, I: 11, C: 11	45%	14	8	Pulmonary function, health-related quality of life.
Del corral T et al. [45], 2017	Spain	*n*: 40, I: 20, C: 20	53%	12	6	Exercise capacity, health-related quality of life, and adverse events.
Schindel C et al. [46], 2015	Brazil	*n*: 34, I: 17, C: 17	59%	13	12	Pulmonary function.
Hommerding P et al. [47], 2015	Brazil	*n*: 34, I: 17, C: 17	59%	13	12	Pulmonary function, exercise capacity, and health-related quality of life.
Eidt P et al. [48], 2014	Brazil	*n*: 41, I: 19, C: 22	34.15%	24	12	Pulmonary function, exercise capacity, and health-related quality of life.
Kriemler S et al. [49], 2013	Switzerland	*n*: 39, I: 29, C: 10	58%	21	24	Pulmonary function, exercise capacity, health-related quality of life, and adverse effects.
Santana et al. [50], 2013	Spain	*n*: 20, I: 10, C: 10	60%	11	8	Pulmonary function, exercise capacity, and adverse events.
Santana et al. [51], 2012	Spain	*n*: 22, I: 11, C: 11	59%	11	8	Pulmonary function, exercise capacity, health-related quality of life, and adverse events.
Sandsund C et al. [52], 2011	UK	*n*: 20, I: 10, C: 10	50%	27	6	Pulmonary function, health-related quality of life, and adverse events.
klijn P et al. [53], 2004	Netherlands	*n*: 20, I: 11, C: 9	NR	14	12	Exercise capacity, health-related quality of life, and adverse events.
Enright S et al. [54], 2004	UK	*n*: 19, I: 9, C: 10	55%	22	8	Pulmonary function and health-related quality of life.
Moorcroft A et al. [55], 2004	UK	*n*: 48, I: 30, C: 18	NR	23	52	Exercise capacity.
Selvadurai et al. [56], 2002	Australia	*n*: 31, I: 15, C: 16	61%	11	8	Pulmonary function, exercise capacity, and adverse events.
Jong W et al. [57], 2001	Netherlands	*n*: 15, I: 7, C: 8	50%	18	6	Pulmonary function and exercise capacity.
Schneiderman J et al. [58], 2000	Canada	*n*: 65, I: 30, C: 35	59%	13	156	Pulmonary function and exercise capacity.
Sawyer H et al. [59], 1993	USA	*n*: 20, I: 10, C: 10	55%	11	10	Pulmonary function.

International: Austria, Canada, France, Germany, Switzerland, Netherlands, United Kingdom, and United States. NR: Not reported.

**Table 2:** Characteristics of the intervention performed in the studies included in the review.

Author, Year	Setting	Program Activities	Number of Sessions	Conclusion
Flores J et al. [37], 2023	Hospital	Early rehabilitation with aerobic exercise on a treadmill + strength.	10	The intervention improved muscle strength, fatigue, and quality of life. No significant differences were found in pulmonary function.
Hebestrit H et al. [38], 2021	Hospital	Vigorous physical activity with motivational feedback. Included 30 min of strength exercises and 2 h of aerobic exercises.	156	The program increased vigorous physical activity and exercise capacity, but the control group showed superior improvement in FEV1 at 6 months.
Kaltsakas G et al. [39], 2021	Hospital	Interval exercise.	36	The intervention improved respiratory muscle strength, with less desaturation and dyspnea during exercise, although it did not improve functional capacity.
Gungor S et al. [40], 2022	Hospital	Respiratory physiotherapy, postural exercises, muscle strengthening, and core stability.	6	The intervention produced no significant changes in respiratory function, exercise tolerance, or postural stability.
Emirza C et al. [41], 2021	Home	Expiratory muscle training with a PEP device.	60	The intervention was related to improvements in PCF, MEP, MIP, 6MWT, and Quality of Life. There were no adverse effects.
Gupta S et al. [42], 2019	Home	Progressive resistance training and plyometric jumping exercises.	144	The intervention improved exercise capacity and quality of life.
Zeren M et al. [43], 2019	Hospital and Home	Chest physiotherapy and inspiratory muscle training.	56	Inspiratory muscle training improved inspiratory strength, with no changes in pulmonary function, exercise tolerance, or quality of life.
Bieli C et al. [44], 2017	Home	Respiratory resistance training with SpiroTiger^®^.	40	The intervention reduced respiratory muscle endurance; however, it did not improve exercise endurance or the other measured outcomes.
Del corral T et al. [45], 2017	Home	Training through active video games (Nintendo Wii^®^).	40	The intervention improved respiratory endurance, with no changes in pulmonary function, tolerance, or quality of life.
Schindel C et al. [46], 2015	Hospital	Aerobic activities, stretching exercises for upper and lower limbs.	39	The intervention did not generate significant changes in pulmonary function.
Hommerding P et al. [47], 2015	Hospital	Verbal and written instructions to perform aerobic activities.	24	The intervention increased the frequency of physical exercise practice reported by participants, but did not generate improvements in pulmonary function, exercise capacity, or quality of life domains.
Eidt P et al. [48], 2014	Home	Daily aerobic and strength exercise supervised by telephone.	84	The intervention improved muscle strength; however, there were no changes in functional capacity or quality of life.
Kriemler S et al. [49], 2013	Home	Strength or endurance training.	72	The treatment improved FEV1 at 6 months. However, the benefits decreased at 24 months without continued training, indicating the importance of long-term regular physical activity.
Santana et al. [50], 2013	Hospital and Home	Inspiratory muscle training, aerobic and strength training.	24	Improvement in exercise capacity and muscle strength. Some benefits were maintained after detraining.
Santana et al. [51], 2012	Hospital	Combination of aerobic exercise on cycle ergometers and circuit weight training.	24	The combined in-hospital strength and aerobic training significantly improved cardiorespiratory capacity and muscle strength in children with cystic fibrosis. However, no improvements were observed in pulmonary function, body composition, or quality of life.
Sandsund C et al. [52], 2011	Hospital	Musculoskeletal treatment, which included mobilizations of the rib cage and thoracic spine.	6	Although no significant short-term changes in pulmonary function were observed, improvements in the thoracic index and rib cage mobility were identified.
klijn P et al. [53], 2004	Hospital	High-intensity anaerobic training.	24	The treatment improved anaerobic performance, aerobic performance, and quality of life.
Enright S et al. [54], 2004	Hospital	Inspiratory muscle training using an electronic manometer.	24	High-intensity training improved strength, lung volumes, and psychosocial status.
Moorcroft A et al. [55], 2004	Home	Individualized and unsupervised home exercise program: Aerobic activities and upper limb strength exercises.	312	The intervention achieved improvements in physical fitness and preserved pulmonary function in adults after 1 year, without the need for continuous supervision.
Selvadurai et al. [56], 2002	Hospital	Aerobic or strength training.	24	The intervention improved exercise capacity and strength. Additionally, an improvement in cardiorespiratory capacity was evidenced.
Jong W et al. [57], 2001	Hospital	Inspiratory muscle training using threshold load devices.	30	The intervention significantly improved inspiratory endurance in patients with cystic fibrosis, but there were no changes in pulmonary function, exercise capacity, dyspnea, or fatigue.
Schneiderman J et al. [58], 2000	Hospital	Aerobic exercise.	468	Regular home aerobic exercise reduced the decline in pulmonary function in patients with cystic fibrosis over 3 years and was feasible to maintain with high adherence.
Sawyer H et al. [59], 1993	Hospital	Inspiratory muscle training using threshold load devices.	70	Inspiratory muscle training significantly improved inspiratory strength, lung capacity, and exercise tolerance.

PEP: Positive Expiratory Pressure; PCF: Peak Cough Flow; MEP: Maximum Expiratory Pressure; MIP: Maximum Inspiratory Pressure.

**Table 3:** Assessment of the quality of the evidence using the Jadad scale.

Author	The Study Is Randomized	The Intervention Is Double-Blind	Study Withdrawals Are Accounted for and Described	The Randomization Procedure Is Adequate	Selection Criteria	Score
Flores J et al. [37], 2023	1	1	1	1	1	5
Hebestrit H et al. [38], 2021	1	0	1	1	1	4
Kaltsakas G et al. [39], 2021	1	0	1	1	1	4
Gungor S et al. [40], 2022	1	0	1	1	1	4
Emirza C et al. [41], 2021	1	1	1	1	1	5
Gupta S et al. [42], 2019	1	0	1	1	1	4
Zeren M et al. [43], 2019	1	0	1	1	1	4
Bieli C et al. [44], 2017	1	0	1	1	1	4
Del corral T et al. [45], 2017	1	0	1	1	1	4
Schindel C et al. [46], 2015	1	0	1	1	1	4
Hommerding P et al. [47], 2015	1	0	1	1	1	4
Eidt P et al. [48], 2014	1	0	1	1	1	4
Kriemler S et al. [49], 2013	1	0	1	1	1	4
Santana et al. [50], 2013	1	0	1	1	1	4
Santana et al. [51], 2012	1	0	1	1	1	4
Sandsund C et al. [52], 2011	1	0	0	1	1	3
klijn P et al. [53], 2004	1	0	1	1	1	4
Enright S et al. [54], 2004	1	1	1	1	1	5
Moorcroft A et al. [55], 2004	1	0	1	1	1	4
Selvadurai et al. [56], 2002	1	0	1	1	1	4
Jong W et al. [57], 2001	1	1	1	1	1	5
Schneiderman J et al. [58], 2000	1	0	1	1	1	4
Sawyer H et al. [59], 1993	1	1	1	0	1	4

**Table 4:** GRADE certainty of evidence for the outcomes.

Outcome	Effect Size (MD or SMD)	GRADE Certainty
Pulmonary function (FEV1)	SMD: 0.05 (−0.09 to 0.20)	⬤⬤◯◯
Pulmonary function (FVC)	SMD: 0.11 (−0.04 to 0.27)	⬤⬤◯◯
Pulmonary function (FEV1/FVC)	MD: 0.04 (−3.07 to 3.15)	⬤⬤◯◯
Pulmonary function (RV/TLC)	SMD: −0.02 (−0.33 to 0.29)	⬤⬤◯◯
Exercise capacity (6MWT)	MD: 20.05 (−0.15 to 40.25)	⬤◯◯◯
Exercise capacity (VO_2_ max)	MD: 2.74 (0.43 to 5.04)	⬤⬤◯◯
Exercise capacity (Wmax)	SMD: 0.05 (−0.17 to 0.28)	⬤⬤◯◯

⬤◯◯◯**: Very low** ⬤⬤◯◯**: Low**.

## Supplementary Materials

The following supporting information can be downloaded at: https://www.mdpi.com/article/10.3390/healthcare13162017/s1; Figure S1: Sensitivity analysis of the effect of pulmonary rehabilitation and physical training on FEV_1_ in patients with cystic fibrosis, modified meta-analysis model; Figure S2: Sensitivity analysis of the effect of pulmonary rehabilitation and physical training on FEV_1_ in patients with cystic fibrosis, with the exclusion of Enright S et al. [51]; Figure S3: Sensitivity analysis of the effect of pulmonary rehabilitation and physical training on FEV_1_ in patients with cystic fibrosis, excluding the study by Kriemler S et al. [46]; Figure S4: Sensitivity analysis of the effect of pulmonary rehabilitation and physical training on FEV_1_ in patients with cystic fibrosis, excluding the study by Selvadurai H et al. [53]; Figure S5: Sensitivity analysis of the effect of pulmonary rehabilitation and physical training on FVC in patients with cystic fibrosis, modified meta-analysis model; Figure S6: Sensitivity analysis of the effect of pulmonary rehabilitation and physical training on FVC in patients with cystic fibrosis, with the exclusion of Enright S et al. [51]; Figure S7: Sensitivity analysis of the effect of pulmonary rehabilitation and physical training on FVC in patients with cystic fibrosis, excluding the study by Kriemler S et al. [46]; Figure S8: Sensitivity analysis of the effect of pulmonary rehabilitation and physical training on FVC in patients with cystic fibrosis, excluding the study by Selvadurai H et al. [53]; Figure S9: Sensitivity analysis of the effect of pulmonary rehabilitation and physical training on FEV_1_/FVC in patients with cystic fibrosis, modified meta-analysis model; Figure S10: Sensitivity analysis of the effect of pulmonary rehabilitation and physical training on RV/TLC in patients with cystic fibrosis, modified meta-analysis model; Figure S11: Subgroup analysis of the 6-minute walking distance according to age group in patients with cystic fibrosis; Figure S12: Subgroup analysis of the 6-minute walking distance according to treatment duration in patients with cystic fibrosis; Figure S13: Sensitivity analysis of the effect of pulmonary rehabilitation and physical training on 6MWD in patients with cystic fibrosis, modified meta-analysis model; Figure S14: Subgroup analysis of the VO_2_ max according to age group in patients with cystic fibrosis; Figure S15: Subgroup analysis of VO_2_ max according to treatment duration in patients with cystic fibrosis; Figure S16: Sensitivity analysis of the effect of pulmonary rehabilitation and physical training on VO_2_ max in patients with cystic fibrosis, excluding the study by Kriemler S et al. [46]; Figure S17: Sensitivity analysis of the effect of pulmonary rehabilitation and physical training on VO_2_ max in patients with cystic fibrosis, excluding the study by Selvadurai H et al. [53]; Figure S18: Sensitivity analysis of the effect of pulmonary rehabilitation and physical training on W max in patients with cystic fibrosis, modified meta-analysis model; Figure S19: Sensitivity analysis of the effect of pulmonary rehabilitation and physical training on W max in patients with cystic fibrosis, excluding the study by Kriemler S et al. [46]. Table S1. Summary of Meta-Analysis Findings: Effects of Physical Training and Pulmonary Rehabilitation on Clinical Outcomes in Patients with Cystic Fibrosis. Table S2. Summary of Findings, Certainty of the evidence.

## Abbreviations

VEF1	Forced Expiratory Volume in 1 s
CVF	Forced Vital Capacity
VEF1/CVF	Ratio of Forced Expiratory Volume in 1 s to Forced Vital Capacity
RV/TLC	Residual Volume to Total Lung Capacity ratio
VO_2_ max	Maximum Oxygen Consumption
6MWT	6-Minute Walk Test
CF	Cystic Fibrosis

## References

[1] Bierlaagh M.C., Muilwijk D., Beekman J.M., van der Ent C.K. (2021). A New Era for People with Cystic Fibrosis. Eur. J. Pediatr..

[2] Calella P., Valerio G., Brodlie M., Donini L.M., Siervo M. (2018). Cystic Fibrosis, Body Composition, and Health Outcomes: A Systematic Review. Nutrition.

[3] Cutting G.R. (2015). Cystic Fibrosis Genetics: From Molecular Understanding to Clinical Application. Nat. Rev. Genet..

[4] Bell S.C., Mall M.A., Gutierrez H., Macek M., Madge S., Davies J.C., Burgel P.-R., Tullis E., Castaños C., Castellani C. (2020). The Future of Cystic Fibrosis Care: A Global Perspective. Lancet Respir. Med..

[5] Varkki S.D., Aaron R., Chapla A., Danda S., Medhi P., Jansi Rani N., Paul G.R. (2024). CFTR Mutations and Phenotypic Correlations in People with Cystic Fibrosis: A Retrospective Study from a Single Centre in South India. Lancet Reg. Health—Southeast Asia.

[6] Polgreen P.M., Comellas A.P. (2022). Clinical Phenotypes of Cystic Fibrosis Carriers. Annu. Rev. Med..

[7] Farinha C.M., Callebaut I. (2022). Molecular Mechanisms of Cystic Fibrosis—How Mutations Lead to Misfunction and Guide Therapy. Biosci. Rep..

[8] Fanen P., Wohlhuter-Haddad A., Hinzpeter A. (2014). Genetics of Cystic Fibrosis: CFTR Mutation Classifications toward Genotype-Based CF Therapies. Int. J. Biochem. Cell Biol..

[9] Lewis B.W., Patial S., Saini Y. (2019). Immunopathology of Airway Surface Liquid Dehydration Disease. J. Immunol. Res..

[10] Ramananda Y., Naren A.P., Arora K. (2024). Functional Consequences of CFTR Interactions in Cystic Fibrosis. Int. J. Mol. Sci..

[11] Fonseca C., Bicker J., Alves G., Falcão A., Fortuna A. (2020). Cystic Fibrosis: Physiopathology and the Latest Pharmacological Treatments. Pharmacol. Res..

[12] Fuhrer M., Zampoli M., Abriel H. (2024). Diagnosing Cystic Fibrosis in Low- and Middle-Income Countries: Challenges and Strategies. Orphanet J. Rare Dis..

[13] Spoonhower K.A., Davis P.B. (2016). Epidemiology of Cystic Fibrosis. Clin. Chest Med..

[14] Scotet V., L’Hostis C., Férec C. (2020). The Changing Epidemiology of Cystic Fibrosis: Incidence, Survival and Impact of the CFTR Gene Discovery. Genes.

[15] Audrézet M.P., Munck A., Scotet V., Claustres M., Roussey M., Delmas D., Férec C., Desgeorges M. (2015). Comprehensive CFTR Gene Analysis of the French Cystic Fibrosis Screened Newborn Cohort: Implications for Diagnosis, Genetic Counseling, and Mutation-Specific Therapy. Genet. Med..

[16] Skov M., Bækvad-Hansen M., Hougaard D.M., Skogstrand K., Lund A.M., Pressler T., Olesen H.V., Duno M. (2020). Cystic Fibrosis Newborn Screening in Denmark: Experience from the First 2 Years. Pediatr. Pulmonol..

[17] Lilley M., Christian S., Hume S., Scott P., Montgomery M., Semple L., Zuberbuhler P., Tabak J., Bamforth F., Somerville M.J. (2010). Newborn Screening for Cystic Fibrosis in Alberta: Two Years of Experience. Paediatr. Child Health.

[18] Kosorok M.R., Wei W.-H., Farrell P.M. (1996). THE INCIDENCE OF CYSTIC FIBROSIS. Statist. Med..

[19] Silva Filho L.V.R.F., Castaños C., Ruíz H.H. (2016). Cystic Fibrosis in Latin America—Improving the Awareness. J. Cyst. Fibros..

[20] Fajac I., Burgel P.-R. (2016). Croissance démographique et thérapeutiques ciblées: Le nouveau visage de la mucoviscidose. Rev. Des Mal. Respir..

[21] Corriveau S., Sykes J., Stephenson A.L. (2018). Cystic Fibrosis Survival: The Changing Epidemiology. Curr. Opin. Pulm. Med..

[22] Stephenson A.L., Stanojevic S., Sykes J., Burgel P.-R. (2017). The Changing Epidemiology and Demography of Cystic Fibrosis. La Presse Médicale.

[23] Garcia B., Flume P.A. (2019). Pulmonary Complications of Cystic Fibrosis. Semin. Respir. Crit. Care Med..

[24] Graeber S.Y., Mall M.A. (2023). The Future of Cystic Fibrosis Treatment: From Disease Mechanisms to Novel Therapeutic Approaches. Lancet.

[25] Connett G. (2019). Lumacaftor-Ivacaftor in the Treatment of Cystic Fibrosis: Design, Development and Place in Therapy. Drug Des. Dev. Ther..

[26] Braga S.F.F., Almgren M.M. (2013). Complementary Therapies in Cystic Fibrosis: Nutritional Supplements and Herbal Products. J. Pharm. Pract..

[27] Lonabaugh K.P., O’Neal K.S., McIntosh H., Condren M. (2018). Cystic Fibrosis-Related Education: Are We Meeting Patient and Caregiver Expectations?. Patient Educ. Couns..

[28] Cruz Mosquera F.E., Perlaza C.L., Naranjo Rojas A., Murillo Rios S., Carrero Gallego A., Fischersworring S.I., Rodríguez J.S., Liscano Y. (2025). Effectiveness of Probiotics, Prebiotics, and Symbiotic Supplementation in Cystic Fibrosis Patients: A Systematic Review and Meta-Analysis of Clinical Trials. Medicina.

[29] Kalamara E.I., Ballas E.T., Pitsiou G., Petrova G. (2021). Pulmonary Rehabilitation for Cystic Fibrosis: A Narrative Review of Current Literature. Monaldi Arch. Chest Dis..

[30] Ward N., Stiller K., Holland A.E. (2019). Exercise as a Therapeutic Intervention for People with Cystic Fibrosis. Expert Rev. Respir. Med..

[31] Schmidt A.M., Jacobsen U., Bregnballe V., Olesen H.V., Ingemann-Hansen T., Thastum M., Oluf Schiøtz P. (2011). Exercise and Quality of Life in Patients with Cystic Fibrosis: A 12-Week Intervention Study. Physiother. Theory Pract..

[32] Hulzebos H.J., Snieder H., Van Der Et J., Helders P.J., Takken T. (2011). High-Intensity Interval Training in an Adolescent with Cystic Fibrosis: A Physiological Perspective. Physiother. Theory Pract..

[33] Gagulic S., Bártolo A., Marques A. (2024). Effects of a Tailored Home-Based Exercise Program, “KidMove”, on Children with Cystic Fibrosis: A Quasi-Experimental Study. Healthcare.

[34] García-Pérez-de-Sevilla G., Yvert T., Blanco Á., Sosa Pedreschi A.I., Thuissard I.J., Pérez-Ruiz M. (2022). Effectiveness of Physical Exercise Interventions on Pulmonary Function and Physical Fitness in Children and Adults with Cystic Fibrosis: A Systematic Review with Meta-Analysis. Healthcare.

[35] Knobloch K., Yoon U., Vogt P.M. (2011). Preferred Reporting Items for Systematic Reviews and Meta-Analyses (PRISMA) Statement and Publication Bias. J. Cranio-Maxillofac. Surg..

[36] Higgins J.P.T., Altman D.G., Gotzsche P.C., Juni P., Moher D., Oxman A.D., Savovic J., Schulz K.F., Weeks L., Sterne J.A.C. (2011). The Cochrane Collaboration’s Tool for Assessing Risk of Bias in Randomised Trials. BMJ.

[37] Flores J., Ziegler B., Silvello D., Dalcin P.T.R. (2023). Effects of an Early Rehabilitation Program for Adult Cystic Fibrosis Patients during Hospitalization: A Randomized Clinical Trial. Braz. J. Med. Biol. Res..

[38] Hebestreit H., Kriemler S., Schindler C., Stein L., Karila C., Urquhart D.S., Orenstein D.M., Lands L.C., Schaeff J., Eber E. (2022). Effects of a Partially Supervised Conditioning Program in Cystic Fibrosis: An International Multicenter, Randomized Controlled Trial (ACTIVATE-CF). Am. J. Respir. Crit. Care Med..

[39] Kaltsakas G., Chynkiamis N., Anastasopoulos N., Zeliou P., Karapatoucha V., Kotsifas K., Diamantea F., Inglezos I., Koulouris N.G., Vogiatzis I. (2021). Interval versus Constant-Load Exercise Training in Adults with Cystic Fibrosis. Respir. Physiol. Neurobiol..

[40] Güngör S. (2021). The Clinical Effects of Combining Postural Exercises with Chest Physiotherapy in Cystic Fibrosis: A Single-Blind, Randomized-Controlled Trial. Turk. J. Phys. Med. Rehabil..

[41] Emirza C., Aslan G.K., Kilinc A.A., Cokugras H. (2021). Effect of Expiratory Muscle Training on Peak Cough Flow in Children and Adolescents with Cystic Fibrosis: A Randomized Controlled Trial. Pediatr. Pulmonol..

[42] Gupta S., Mukherjee A., Lodha R., Kabra M., Deepak K.K., Khadgawat R., Talwar A., Kabra S.K. (2019). Effects of Exercise Intervention Program on Bone Mineral Accretion in Children and Adolescents with Cystic Fibrosis: A Randomized Controlled Trial. Indian J. Pediatr..

[43] Zeren M., Cakir E., Gurses H.N. (2019). Effects of Inspiratory Muscle Training on Postural Stability, Pulmonary Function and Functional Capacity in Children with Cystic Fibrosis: A Randomised Controlled Trial. Respir. Med..

[44] Bieli C., Summermatter S., Boutellier U., Moeller A. (2017). Respiratory Muscle Training Improves Respiratory Muscle Endurance but Not Exercise Tolerance in Children with Cystic Fibrosis. Pediatr. Pulmonol..

[45] Del Corral T., Cebrià I Iranzo M.À., López-de-Uralde-Villanueva I., Martínez-Alejos R., Blanco I., Vilaró J. (2018). Effectiveness of a Home-Based Active Video Game Programme in Young Cystic Fibrosis Patients. Respiration.

[46] Schindel C.S., Hommerding P.X., Melo D.A.S., Baptista R.R., Marostica P.J.C., Donadio M.V.F. (2015). Physical Exercise Recommendations Improve Postural Changes Found in Children and Adolescents with Cystic Fibrosis: A Randomized Controlled Trial. J. Pediatr..

[47] Hommerding P.X., Baptista R.R., Makarewicz G.T., Schindel C.S., Donadio M.V., Pinto L.A., Marostica P.J. (2015). Effects of an Educational Intervention of Physical Activity for Children and Adolescents With Cystic Fibrosis: A Randomized Controlled Trial. Respir. Care.

[48] Eidt P.M., Flores J., Ziegler B., Casarotto F., Jaques P., Barreto S.S.M., Dalcin P.D.T.R. (2014). Exercise Programme in Patients with Cystic Fibrosis: A Randomized Controlled Trial. Respir. Med..

[49] Kriemler S., Kieser S., Junge S., Ballmann M., Hebestreit A., Schindler C., Stüssi C., Hebestreit H. (2013). Effect of Supervised Training on FEV1 in Cystic Fibrosis: A Randomised Controlled Trial. J. Cyst. Fibros..

[50] Santana E., Gonzalez-Saiz L., Groeneveld I.F., Villa-Asensi J.R., Barrio Gómez De Aguero M.I., Fleck S.J., López-Mojares L.M., Pérez M., Lucia A. (2014). Benefits of Combining Inspiratory Muscle with ‘Whole Muscle’ Training in Children with Cystic Fibrosis: A Randomised Controlled Trial. Br. J. Sports Med..

[51] Santana E., Groeneveld I.F., Gonzalez-Saiz L., López-Mojares L.M., Villa-Asensi J.R., Gonzalez M.I.B., Fleck S.J., Pérez M., Lucia A. (2012). Intrahospital Weight and Aerobic Training in Children with Cystic Fibrosis: A Randomized Controlled Trial. Med. Sci. Sports Exerc..

[52] Sandsund C.A., Roughton M., Hodson M.E., Pryor J.A. (2011). Musculoskeletal Techniques for Clinically Stable Adults with Cystic Fibrosis: A Preliminary Randomised Controlled Trial. Physiotherapy.

[53] Klijn P.H.C., Oudshoorn A., Van Der Ent C.K., Van Der Net J., Kimpen J.L., Helders P.J.M. (2004). Effects of Anaerobic Training in Children With Cystic Fibrosis. Chest.

[54] Enright S., Chatham K., Ionescu A.A., Unnithan V.B., Shale D.J. (2004). Inspiratory Muscle Training Improves Lung Function and Exercise Capacity in Adults With Cystic Fibrosis. Chest.

[55] Moorcroft A.J. (2004). Individualised Unsupervised Exercise Training in Adults with Cystic Fibrosis: A 1 Year Randomised Controlled Trial. Thorax.

[56] Selvadurai H.C., Blimkie C.J., Meyers N., Mellis C.M., Cooper P.J., Van Asperen P.P. (2002). Randomized Controlled Study of Inhospital Exercise Training Programs in Children with Cystic Fibrosis. Pediatr. Pulmonol..

[57] De Jong W., Van Aalderen W.M.C., Kraan J., Koëter G.H., Van Der Schans C.P. (2001). Inspiratory Muscle Training in Patients with Cystic Fibrosis. Respir. Med..

[58] Schneiderman J., Pollock S.L., Corey M., Wilkes D.D., Canny G.J., Pedder L., Reisman J.J. (2000). A Randomized Controlled Trial of a 3-Year Home Exercise Program in Cystic Fibrosis. J. Pediatr..

[59] Sawyer E.H., Clanton T.L. (1993). Improved Pulmonary Function and Exercise Tolerance With Inspiratory Muscle Conditioning in Children With Cystic Fibrosis. Chest.

[60] da Silva F.C., Arancibia B.A.V., Iop R.d.R., Filho P.J.B.G., Silva R.d. (2013). Escalas y listas de evaluación de la calidad de estudios científicos. Rev. Cuba. De Inf. En Cienc. De La Salud (ACIMED).

[61] Granholm A., Alhazzani W., Møller M.H. (2019). Use of the GRADE Approach in Systematic Reviews and Guidelines. Br. J. Anaesth..

[62] Cai W., Li M., Xu Y., Li M., Wang J., Zuo Y., Cao J. (2024). The Effect of Respiratory Muscle Training on Children and Adolescents with Cystic Fibrosis: A Systematic Review and Meta-Analysis. BMC Pediatr..

[63] Stanford G., Ryan H., Solis-Moya A. (2020). Respiratory Muscle Training for Cystic Fibrosis. Cochrane Database Syst. Rev..

[64] Radtke T., Nevitt S.J., Hebestreit H., Kriemler S. (2017). Physical Exercise Training for Cystic Fibrosis. Cochrane Database Syst. Rev..

[65] Van Doorn N. (2010). Exercise Programs for Children with Cystic Fibrosis: A Systematic Review of Randomized Controlled Trials. Disabil. Rehabil..

[66] Gruber W., Stehling F., Blosch C., Dillenhoefer S., Olivier M., Koerner-Rettberg C., Sutharsan S., Mellies U., Taube C., Welsner M. (2022). Effects of a Long-Term Monitored Exercise Program on Aerobic Fitness in a Small Group of Children with Cystic Fibrosis. Int. J. Environ. Res. Public Health.

[67] Rosolia Capasso C., Miniato A.L., Di Filippo P., Di Ludovico A., Di Pillo S., Chiarelli F., Sferrazza Papa G.F., Attanasi M. (2025). The Impact of Physical Activity on Clinical Outcomes in Children with Cystic Fibrosis: A Narrative Review. Children.

[68] Hilliard T.N., Regamey N., Shute J.K., Nicholson A.G., Alton E.W.F.W., Bush A., Davies J.C. (2007). Airway Remodelling in Children with Cystic Fibrosis. Thorax.

[69] Levine H., Cohen-Cymberknoh M., Klein N., Hoshen M., Mussaffi H., Stafler P., Breuer O., Kerem E., Blau H. (2016). Reversible Airway Obstruction in Cystic Fibrosis: Common, but Not Associated with Characteristics of Asthma. J. Cyst. Fibros..

[70] Pastré J., Prévotat A., Tardif C., Langlois C., Duhamel A., Wallaert B. (2014). Determinants of Exercise Capacity in Cystic Fibrosis Patients with Mild-to-Moderate Lung Disease. BMC Pulm. Med..

[71] Pianosi P. (2005). Peak Oxygen Uptake and Mortality in Children with Cystic Fibrosis. Thorax.

[72] De Jong W., Grevink R.G., Roorda R.J., Kaptein A.A., Van Der Schans C.P. (1994). Effect of a Home Exercise Training Program in Patients With Cystic Fibrosis. Chest.

[73] Reuveny R., DiMenna F.J., Gunaratnam C., Arad A.D., McElvaney G.N., Susta D., Peled M., Moyna N.M. (2020). High-Intensity Interval Training Accelerates Oxygen Uptake Kinetics and Improves Exercise Tolerance for Individuals with Cystic Fibrosis. BMC Sports Sci. Med. Rehabil..

[74] Hackshaw A. (2008). Small Studies: Strengths and Limitations. Eur. Respir. J..

[75] Cruz Mosquera F.E., Murillo S.R., Naranjo Rojas A., Perlaza C.L., Castro Osorio D., Liscano Y. (2024). Effect of Exercise and Pulmonary Rehabilitation in Pre- and Post-Surgical Patients with Lung Cancer: Systematic Review and Meta-Analysis. Medicina.

[76] Da Silva Filho L.V.R.F., Zampoli M., Cohen-Cymberknoh M., Kabra S.K. (2021). Cystic Fibrosis in Low and Middle-Income Countries (LMIC): A View from Four Different Regions of the World. Paediatr. Respir. Rev..

