# Supplementary material for: Physical Training and Pulmonary Rehabilitation in Patients with Cystic Fibrosis: A Systematic Review and Meta-Analysis of Clinical Trials
Source: Healthcare (Basel). 2025 Aug 15;13(16):2017. doi: 10.3390/healthcare13162017 (PMC12385196; doi:10.3390/healthcare13162017)
Supplement: Supplementary file 1 [file healthcare-13-02017-s001.zip › Figure S1-19.pdf]

Figure S1. Sensitivity analysis of the effect of pulmonary rehabilitation and physical training on FEV_1 in patients with cystic fibrosis, modified meta-analysis model.

Study or Subgroup	Experimental Mean	Experimental SD	Experimental Total	Control Mean	Control SD	Control Total	Weight	Std. mean difference IV, Random, 95% CI
✓ Bieli C et al. 2017	1.48	2.17	11	1.62	2.11	11	3.3%	-0.06 [-0.90 , 0.77]
✓ Eidt P et al. 2014	6	16.1	19	2	7.3	22	5.7%	0.32 [-0.30 , 0.94]
✓ Emirza C et al. 2020	0.14	9.37	14	2.5	17.7	14	4.1%	-0.16 [-0.90 , 0.58]
✓ Enright S et al. 2004	2	1	9	2	1	10	2.8%	0.00 [-0.90 , 0.90]
✓ Flores J et al. 2023	43.1	14.5	19	40.9	13.4	15	4.8%	0.15 [-0.52 , 0.83]
✓ Gungor S et al. 2021	88.5	6.4	10	95.5	36.4	9	2.8%	-0.26 [-1.17 , 0.64]
✓ Gupta S et al.2017	57.5	24.06	25	58.9	22.16	27	7.1%	-0.06 [-0.60 , 0.48]
✓ Hebestrit et al. 2022	75.6	26.6	60	75.6	23.7	57	13.8%	0.00 [-0.36 , 0.36]
✓ Hommerding P et al. 2015	1.8	8.6	17	-1	14.2	17	4.8%	0.23 [-0.44 , 0.91]
✓ Jong W et al. 2001	2.33	0.71	8	2.56	1.09	8	2.4%	-0.24 [-1.22 , 0.75]
✓ Kriemler S et al 2013	0.31	13.2	17	12.1	12	10	3.4%	-0.89 [-1.72 , -0.07]
✓ Sandsun et al. 2011	1.79	0.2	10	1.8	0.13	10	3.0%	-0.06 [-0.93 , 0.82]
✓ Santana E et al. 2012	1.94	0.23	55	1.87	0.15	64	13.8%	0.36 [0.00 , 0.73]
✓ Santana E et al. 2013	1.74	0.23	10	1.55	0.26	10	2.8%	0.74 [-0.17 , 1.65]
✓ Sawyer H et al. 1993	1.96	0.86	10	1.41	0.63	10	2.8%	0.70 [-0.21 , 1.61]
✓ Scheneiderman J et al. 2000	1.46	3.55	30	3.47	4.9	35	8.4%	-0.46 [-0.95 , 0.04]
✓ Schindel C et al. 2015	1.8	8.6	17	-2.7	12.8	17	4.8%	0.40 [-0.28 , 1.08]
✓ Selvadurai H et al. 2002	0.29	7.28	15	-0.21	7.03	16	4.5%	0.07 [-0.64 , 0.77]
✓ Zeren M et al. 2019	3.36	4.39	18	3.69	4.42	18	5.1%	-0.07 [-0.73 , 0.58]
**Total (Wald[a])**			**374**			**380**	**100.0%**	**0.05 [-0.11 , 0.20]**

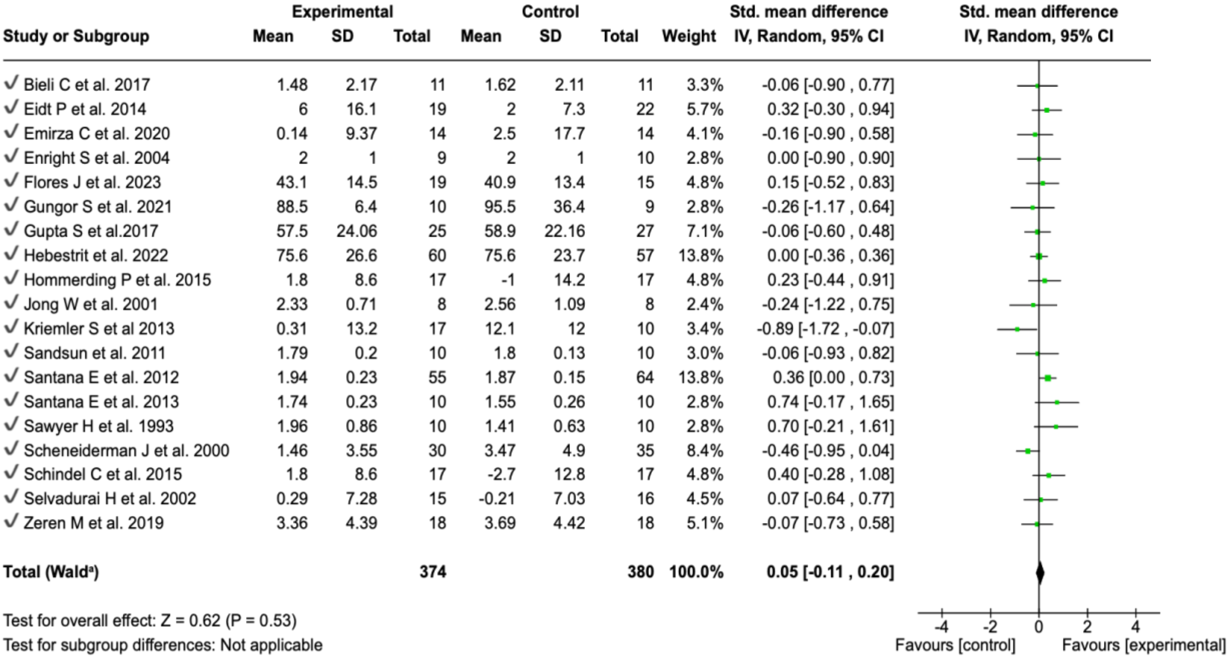

Test for overall effect: Z = 0.62 (P = 0.53)
Test for subgroup differences: Not applicable
Heterogeneity: Tau² (DL[b]) = 0.01; Chi² = 19.90, df = 18 (P = 0.34); I² = 10%

Figure S2. Sensitivity analysis of the effect of pulmonary rehabilitation and physical training on FEV_1 in patients with cystic fibrosis, with the exclusion of Enright S et al. [53]

Study or Subgroup	Experimental Mean	Experimental SD	Experimental Total	Control Mean	Control SD	Control Total	Weight	Std. mean difference IV, Fixed, 95% CI
✓ Bieli C et al. 2017	1.48	2.17	11	1.62	2.11	11	3.1%	-0.06 [-0.90 , 0.77]
✓ Eidt P et al. 2014	6	16.1	19	2	7.3	22	5.6%	0.32 [-0.30 , 0.94]
✓ Emirza C et al. 2020	0.14	9.37	14	2.5	17.7	14	3.9%	-0.16 [-0.90 , 0.58]
× Enright S et al. 2004	2	1	9	2	1	10	0.0%	0.00 [-0.90 , 0.90]
✓ Flores J et al. 2023	43.1	14.5	19	40.9	13.4	15	4.6%	0.15 [-0.52 , 0.83]
✓ Gungor S et al. 2021	88.5	6.4	10	95.5	36.4	9	2.6%	-0.26 [-1.17 , 0.64]
✓ Gupta S et al.2017	57.5	24.06	25	58.9	22.16	27	7.2%	-0.06 [-0.60 , 0.48]
✓ Hebestrit et al. 2022	75.6	26.6	60	75.6	23.7	57	16.2%	0.00 [-0.36 , 0.36]
✓ Hommerding P et al. 2015	1.8	8.6	17	-1	14.2	17	4.7%	0.23 [-0.44 , 0.91]
✓ Jong W et al. 2001	2.33	0.71	8	2.56	1.09	8	2.2%	-0.24 [-1.22 , 0.75]
✓ Kriemler S et al 2013	0.31	13.2	17	12.1	12	10	3.2%	-0.89 [-1.72 , -0.07]
✓ Sandsun et al. 2011	1.79	0.2	10	1.8	0.13	10	2.8%	-0.06 [-0.93 , 0.82]
✓ Santana E et al. 2012	1.94	0.23	55	1.87	0.15	64	16.2%	0.36 [0.00 , 0.73]
✓ Santana E et al. 2013	1.74	0.23	10	1.55	0.26	10	2.6%	0.74 [-0.17 , 1.65]
✓ Sawyer H et al. 1993	1.96	0.86	10	1.41	0.63	10	2.6%	0.70 [-0.21 , 1.61]
✓ Scheneiderman J et al. 2000	1.46	3.55	30	3.47	4.9	35	8.7%	-0.46 [-0.95 , 0.04]
✓ Schindel C et al. 2015	1.8	8.6	17	-2.7	12.8	17	4.6%	0.40 [-0.28 , 1.08]
✓ Selvadurai H et al. 2002	0.29	7.28	15	-0.21	7.03	16	4.3%	0.07 [-0.64 , 0.77]
✓ Zeren M et al. 2019	3.36	4.39	18	3.69	4.42	18	5.0%	-0.07 [-0.73 , 0.58]
**Total**			**365**			**370**	**100.0%**	**0.06 [-0.09 , 0.20]**

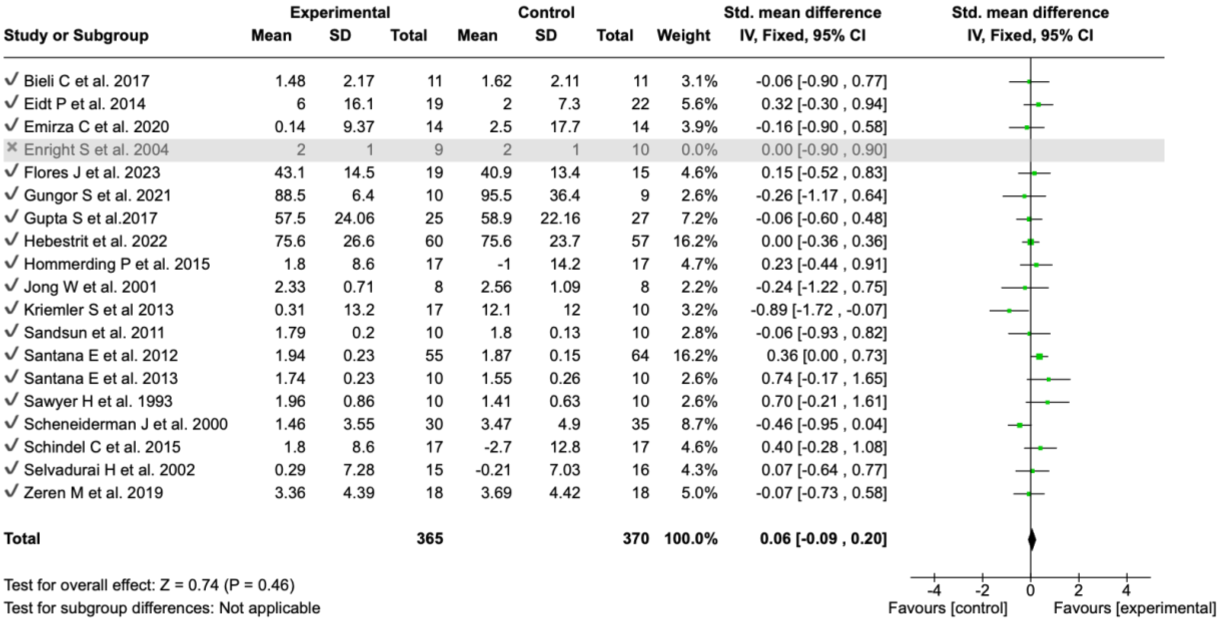

Test for overall effect: Z = 0.74 (P = 0.46)
Test for subgroup differences: Not applicable
Heterogeneity: Chi² = 19.89, df = 17 (P = 0.28); I² = 15%

Figure S3. Sensitivity analysis of the effect of pulmonary rehabilitation and physical training on FEV$_1$ in patients with cystic fibrosis, excluding the study by Kriemler S et al. [48]

Study or Subgroup	Experimental Mean	Experimental SD	Experimental Total	Control Mean	Control SD	Control Total	Weight	Std. mean difference IV, Fixed, 95% CI
✓ Bieli C et al. 2017	1.48	2.17	11	1.62	2.11	11	3.1%	-0.06 [-0.90 , 0.77]
✓ Eidt P et al. 2014	6	16.1	19	2	7.3	22	5.6%	0.32 [-0.30 , 0.94]
✓ Emirza C et al. 2020	0.14	9.37	14	2.5	17.7	14	3.9%	-0.16 [-0.90 , 0.58]
✓ Enright S et al. 2004	2	1	9	2	1	10	2.6%	0.00 [-0.90 , 0.90]
✓ Flores J et al. 2023	43.1	14.5	19	40.9	13.4	15	4.7%	0.15 [-0.52 , 0.83]
✓ Gungor S et al. 2021	88.5	6.4	10	95.5	36.4	9	2.6%	-0.26 [-1.17 , 0.64]
✓ Gupta S et al.2017	57.5	24.06	25	58.9	22.16	27	7.2%	-0.06 [-0.60 , 0.48]
✓ Hebestrit et al. 2022	75.6	26.6	60	75.6	23.7	57	16.3%	0.00 [-0.36 , 0.36]
✓ Hommerding P et al. 2015	1.8	8.6	17	-1	14.2	17	4.7%	0.23 [-0.44 , 0.91]
✓ Jong W et al. 2001	2.33	0.71	8	2.56	1.09	8	2.2%	-0.24 [-1.22 , 0.75]
× Kriemler S et al 2013	0.31	13.2	17	12.1	12	10	0.0%	-0.89 [-1.72 , -0.07]
✓ Sandsun et al. 2011	1.79	0.2	10	1.8	0.13	10	2.8%	-0.06 [-0.93 , 0.82]
✓ Santana E et al. 2012	1.94	0.23	55	1.87	0.15	64	16.2%	0.36 [0.00 , 0.73]
✓ Santana E et al. 2013	1.74	0.23	10	1.55	0.26	10	2.6%	0.74 [-0.17 , 1.65]
✓ Sawyer H et al. 1993	1.96	0.86	10	1.41	0.63	10	2.6%	0.70 [-0.21 , 1.61]
✓ Scheneiderman J et al. 2000	1.46	3.55	30	3.47	4.9	35	8.8%	-0.46 [-0.95 , 0.04]
✓ Schindel C et al. 2015	1.8	8.6	17	-2.7	12.8	17	4.6%	0.40 [-0.28 , 1.08]
✓ Selvadurai H et al. 2002	0.29	7.28	15	-0.21	7.03	16	4.3%	0.07 [-0.64 , 0.77]
✓ Zeren M et al. 2019	3.36	4.39	18	3.69	4.42	18	5.0%	-0.07 [-0.73 , 0.58]
**Total**			**357**			**370**	**100.0%**	**0.08 [-0.06 , 0.23]**

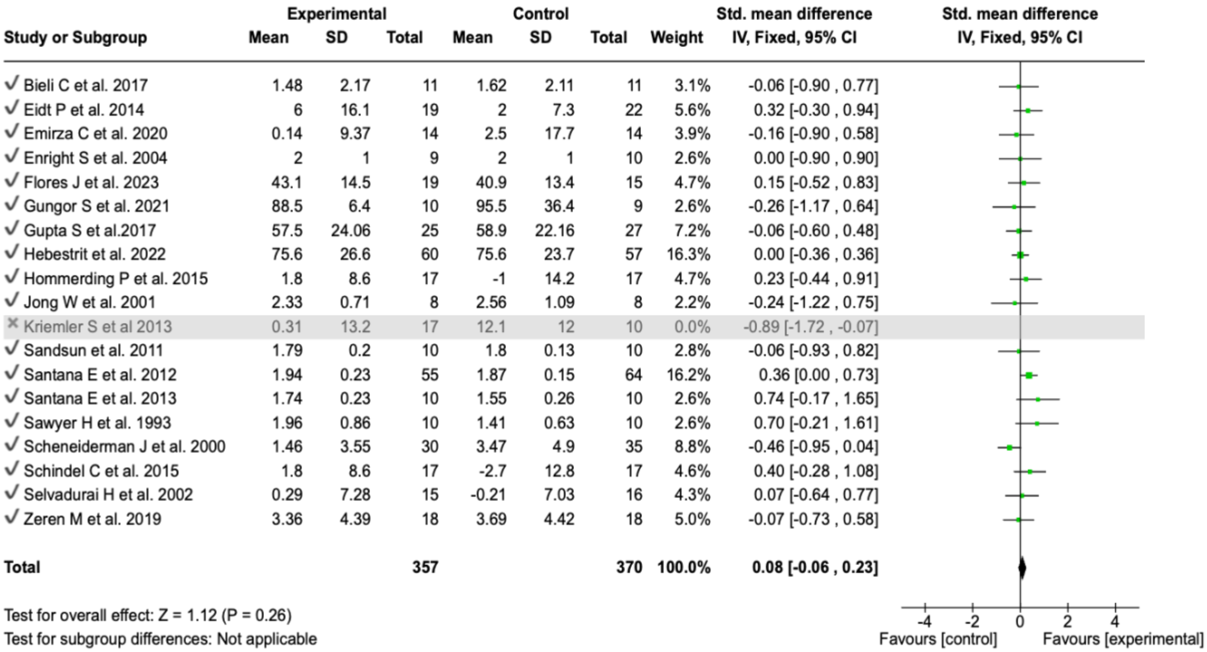

Test for overall effect: Z = 1.12 (P = 0.26)
Test for subgroup differences: Not applicable
Heterogeneity: Chi² = 14.64, df = 17 (P = 0.62); I² = 0%

Figure S4. Sensitivity analysis of the effect of pulmonary rehabilitation and physical training on FEV$_1$ in patients with cystic fibrosis, excluding the study by Selvadurai H et al. [55]

Study or Subgroup	Experimental Mean	Experimental SD	Experimental Total	Control Mean	Control SD	Control Total	Weight	Std. mean difference IV, Fixed, 95% CI
✓ Bieli C et al. 2017	1.48	2.17	11	1.62	2.11	11	3.1%	-0.06 [-0.90 , 0.77]
✓ Eidt P et al. 2014	6	16.1	19	2	7.3	22	5.7%	0.32 [-0.30 , 0.94]
✓ Emirza C et al. 2020	0.14	9.37	14	2.5	17.7	14	3.9%	-0.16 [-0.90 , 0.58]
✓ Enright S et al. 2004	2	1	9	2	1	10	2.7%	0.00 [-0.90 , 0.90]
✓ Flores J et al. 2023	43.1	14.5	19	40.9	13.4	15	4.7%	0.15 [-0.52 , 0.83]
✓ Gungor S et al. 2021	88.5	6.4	10	95.5	36.4	9	2.6%	-0.26 [-1.17 , 0.64]
✓ Gupta S et al.2017	57.5	24.06	25	58.9	22.16	27	7.3%	-0.06 [-0.60 , 0.48]
✓ Hebestrit et al. 2022	75.6	26.6	60	75.6	23.7	57	16.5%	0.00 [-0.36 , 0.36]
✓ Hommerding P et al. 2015	1.8	8.6	17	-1	14.2	17	4.8%	0.23 [-0.44 , 0.91]
✓ Jong W et al. 2001	2.33	0.71	8	2.56	1.09	8	2.2%	-0.24 [-1.22 , 0.75]
✓ Kriemler S et al 2013	0.31	13.2	17	12.1	12	10	3.2%	-0.89 [-1.72 , -0.07]
✓ Sandsun et al. 2011	1.79	0.2	10	1.8	0.13	10	2.8%	-0.06 [-0.93 , 0.82]
✓ Santana E et al. 2012	1.94	0.23	55	1.87	0.15	64	16.4%	0.36 [0.00 , 0.73]
✓ Santana E et al. 2013	1.74	0.23	10	1.55	0.26	10	2.6%	0.74 [-0.17 , 1.65]
✓ Sawyer H et al. 1993	1.96	0.86	10	1.41	0.63	10	2.6%	0.70 [-0.21 , 1.61]
✓ Scheneiderman J et al. 2000	1.46	3.55	30	3.47	4.9	35	8.9%	-0.46 [-0.95 , 0.04]
✓ Schindel C et al. 2015	1.8	8.6	17	-2.7	12.8	17	4.7%	0.40 [-0.28 , 1.08]
× Selvadurai H et al. 2002	0.29	7.28	15	-0.21	7.03	16	0.0%	0.07 [-0.64 , 0.77]
✓ Zeren M et al. 2019	3.36	4.39	18	3.69	4.42	18	5.1%	-0.07 [-0.73 , 0.58]
**Total**			**359**			**364**	**100.0%**	**0.05 [-0.09 , 0.20]**

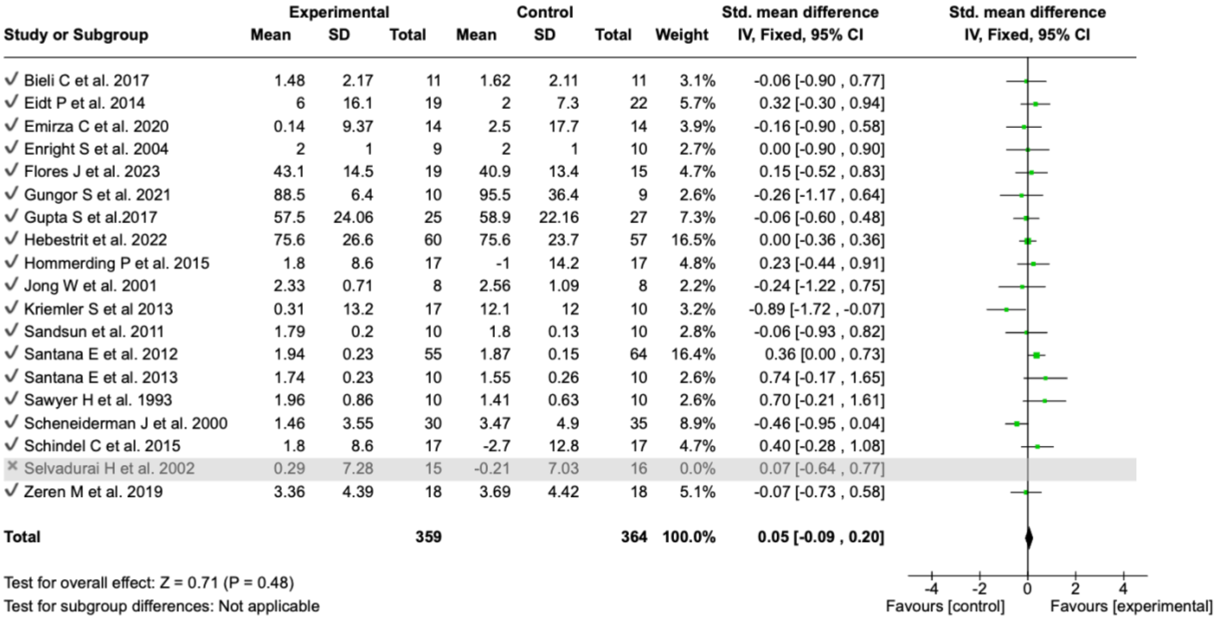

Test for overall effect: Z = 0.71 (P = 0.48)
Test for subgroup differences: Not applicable
Heterogeneity: Chi² = 19.90, df = 17 (P = 0.28); I² = 15%

Figure S5. Sensitivity analysis of the effect of pulmonary rehabilitation and physical training on FVC in patients with cystic fibrosis, modified meta-analysis model.

Study or Subgroup	Experimental Mean [L,mL,%]	Experimental SD [L,mL,%]	Experimental Total	Control Mean [L,mL,%]	Control SD [L,mL,%]	Control Total	Weight	Std. mean difference IV, Random, 95% CI [L,mL,%]
√ Bieli C et al. 2017	1.1	1.8	11	1.17	1.81	11	4.6%	-0.04 [-0.87 , 0.80]
√ Eidt P et al. 2014	6.8	17.7	19	3.5	8.3	22	6.7%	0.24 [-0.38 , 0.86]
√ Emirza C et al. 2020	1.5	11.9	14	2.14	15.6	14	5.4%	-0.04 [-0.79 , 0.70]
√ Enright S et al. 2004	0	0	0	0	0	0		Not estimable
√ Flores J et al. 2023	55.5	14.7	19	55.7	16.7	15	6.1%	-0.01 [-0.69 , 0.66]
√ Gungor S et al. 2021	93.5	10.3	10	95	30.7	9	4.2%	-0.06 [-0.96 , 0.84]
√ Gupta S et al.2017	71	23.4	25	68	9.21	27	7.7%	0.17 [-0.38 , 0.71]
√ Hebestrit et al. 2022	90.3	22.2	60	88.6	21.3	57	10.5%	0.08 [-0.29 , 0.44]
√ Hommerding P et al. 2015	0.4	6.7	17	-2	12.2	17	6.1%	0.24 [-0.44 , 0.91]
√ Jong W et al. 2001	3.07	0.9	8	3.3	0.95	8	3.6%	-0.24 [-1.22 , 0.75]
√ Kriemler S et al 2013	3.29	12.1	17	12.39	10.6	10	4.8%	-0.76 [-1.57 , 0.05]
√ Santana E et al. 2012	2.49	0.25	55	2.36	0.2	64	10.4%	0.58 [0.21 , 0.94]
√ Santana E et al. 2013	2.34	0.29	10	1.85	0.32	10	3.4%	1.54 [0.51 , 2.56]
√ Scheneiderman J et al. 2000	0.25	2.81	30	2.42	4.1	35	8.3%	-0.60 [-1.10 , -0.10]
√ Schindel C et al. 2015	0.41	6.8	17	-1.8	12.2	17	6.1%	0.22 [-0.46 , 0.89]
√ Selvadurai H et al. 2002	0.14	4.46	15	0.03	4.2	16	5.8%	0.02 [-0.68 , 0.73]
√ Zeren M et al. 2019	3.88	3.46	18	3.13	3.18	18	6.3%	0.22 [-0.43 , 0.88]
**Total (Wald[a])**			**345**			**350**	**100.0%**	**0.09 [-0.13 , 0.31]**

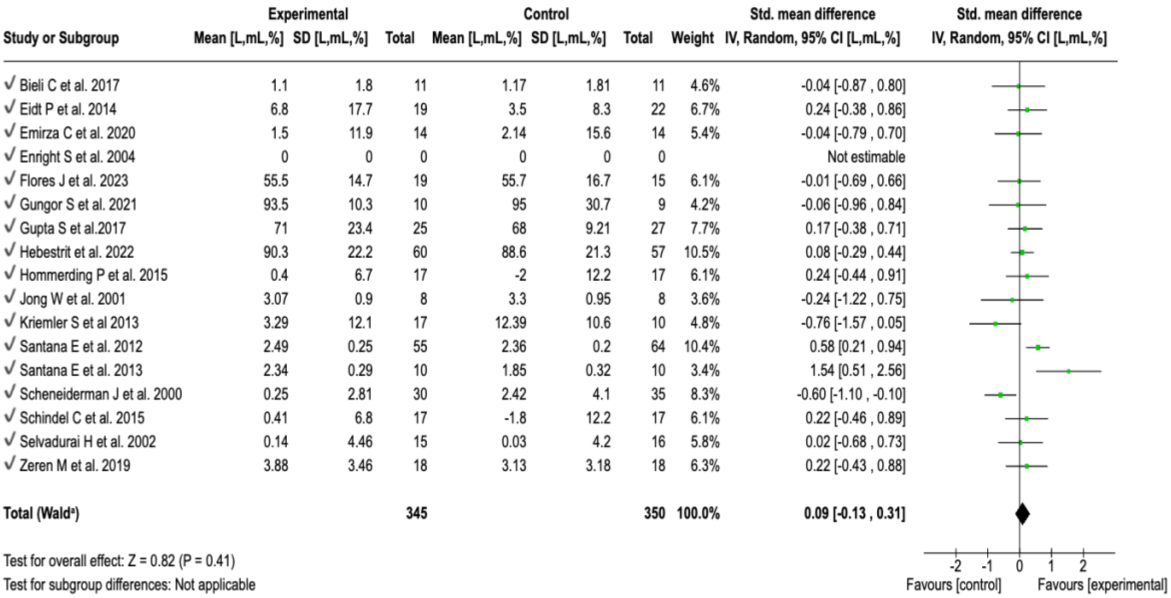

Test for overall effect: Z = 0.82 (P = 0.41)
Test for subgroup differences: Not applicable
Heterogeneity: Tau^2 (DL[b]) = 0.08; Chi^2 = 27.50, df = 15 (P = 0.02); I^2 = 45%

Figure S6. Sensitivity analysis of the effect of pulmonary rehabilitation and physical training on FVC in patients with cystic fibrosis, with the exclusion of Enright S et al. [53]

Study or Subgroup	Experimental Mean [L,mL,%]	Experimental SD [L,mL,%]	Experimental Total	Control Mean [L,mL,%]	Control SD [L,mL,%]	Control Total	Weight	Std. mean difference IV, Fixed, 95% CI [L,mL,%]
√ Bieli C et al. 2017	1.1	1.8	11	1.17	1.81	11	3.3%	-0.04 [-0.87 , 0.80]
√ Eidt P et al. 2014	6.8	17.7	19	3.5	8.3	22	6.0%	0.24 [-0.38 , 0.86]
√ Emirza C et al. 2020	1.5	11.9	14	2.14	15.6	14	4.1%	-0.04 [-0.79 , 0.70]
× Enright S et al. 2004	0	0	0	0	0	0		Not estimable
√ Flores J et al. 2023	55.5	14.7	19	55.7	16.7	15	5.0%	-0.01 [-0.69 , 0.66]
√ Gungor S et al. 2021	93.5	10.3	10	95	30.7	9	2.8%	-0.06 [-0.96 , 0.84]
√ Gupta S et al.2017	71	23.4	25	68	9.21	27	7.7%	0.17 [-0.38 , 0.71]
√ Hebestrit et al. 2022	90.3	22.2	60	88.6	21.3	57	17.3%	0.08 [-0.29 , 0.44]
√ Hommerding P et al. 2015	0.4	6.7	17	-2	12.2	17	5.0%	0.24 [-0.44 , 0.91]
√ Jong W et al. 2001	3.07	0.9	8	3.3	0.95	8	2.4%	-0.24 [-1.22 , 0.75]
√ Kriemler S et al 2013	3.29	12.1	17	12.39	10.6	10	3.5%	-0.76 [-1.57 , 0.05]
√ Santana E et al. 2012	2.49	0.25	55	2.36	0.2	64	16.8%	0.58 [0.21 , 0.94]
√ Santana E et al. 2013	2.34	0.29	10	1.85	0.32	10	2.2%	1.54 [0.51 , 2.56]
√ Scheneiderman J et al. 2000	0.25	2.81	30	2.42	4.1	35	9.1%	-0.60 [-1.10 , -0.10]
√ Schindel C et al. 2015	0.41	6.8	17	-1.8	12.2	17	5.0%	0.22 [-0.46 , 0.89]
√ Selvadurai H et al. 2002	0.14	4.46	15	0.03	4.2	16	4.6%	0.02 [-0.68 , 0.73]
√ Zeren M et al. 2019	3.88	3.46	18	3.13	3.18	18	5.3%	0.22 [-0.43 , 0.88]
**Total**			**345**			**350**	**100.0%**	**0.11 [-0.04 , 0.27]**

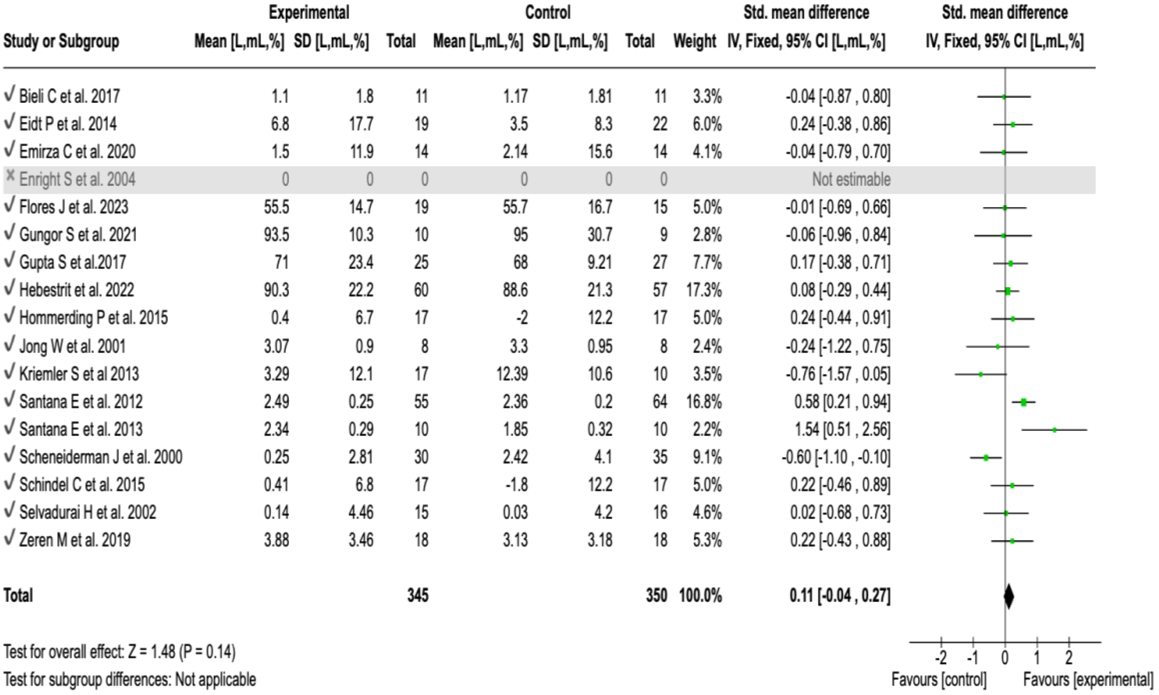

Test for overall effect: Z = 1.48 (P = 0.14)
Test for subgroup differences: Not applicable
Heterogeneity: Chi^2 = 27.50, df = 15 (P = 0.02); I^2 = 45%

Figure S7. Sensitivity analysis of the effect of pulmonary rehabilitation and physical training on FVC in patients with cystic fibrosis, excluding the study by Kriemler S et al. [48]

Study or Subgroup	Experimental Mean [L,mL,%]	Experimental SD [L,mL,%]	Experimental Total	Control Mean [L,mL,%]	Control SD [L,mL,%]	Control Total	Weight	Std. mean difference IV, Fixed, 95% CI [L,mL,%]
√ Bieli C et al. 2017	1.1	1.8	11	1.17	1.81	11	3.4%	-0.04 [-0.87 , 0.80]
√ Eidt P et al. 2014	6.8	17.7	19	3.5	8.3	22	6.2%	0.24 [-0.38 , 0.86]
√ Emirza C et al. 2020	1.5	11.9	14	2.14	15.6	14	4.3%	-0.04 [-0.79 , 0.70]
√ Enright S et al. 2004	0	0	0	0	0	0		Not estimable
√ Flores J et al. 2023	55.5	14.7	19	55.7	16.7	15	5.1%	-0.01 [-0.69 , 0.66]
√ Gungor S et al. 2021	93.5	10.3	10	95	30.7	9	2.9%	-0.06 [-0.96 , 0.84]
√ Gupta S et al.2017	71	23.4	25	68	9.21	27	7.9%	0.17 [-0.38 , 0.71]
√ Hebestrit et al. 2022	90.3	22.2	60	88.6	21.3	57	17.9%	0.08 [-0.29 , 0.44]
√ Hommerding P et al. 2015	0.4	6.7	17	-2	12.2	17	5.2%	0.24 [-0.44 , 0.91]
√ Jong W et al. 2001	3.07	0.9	8	3.3	0.95	8	2.4%	-0.24 [-1.22 , 0.75]
× Kriemler S et al 2013	3.29	12.1	17	12.39	10.6	10	0.0%	-0.76 [-1.57 , 0.05]
√ Santana E et al. 2012	2.49	0.25	55	2.36	0.2	64	17.4%	0.58 [0.21 , 0.94]
√ Santana E et al. 2013	2.34	0.29	10	1.85	0.32	10	2.2%	1.54 [0.51 , 2.56]
√ Scheneiderman J et al. 2000	0.25	2.81	30	2.42	4.1	35	9.5%	-0.60 [-1.10 , -0.10]
√ Schindel C et al. 2015	0.41	6.8	17	-1.8	12.2	17	5.2%	0.22 [-0.46 , 0.89]
√ Selvadurai H et al. 2002	0.14	4.46	15	0.03	4.2	16	4.8%	0.02 [-0.68 , 0.73]
√ Zeren M et al. 2019	3.88	3.46	18	3.13	3.18	18	5.5%	0.22 [-0.43 , 0.88]
**Total**			**328**			**340**	**100.0%**	**0.15 [-0.01 , 0.30]**

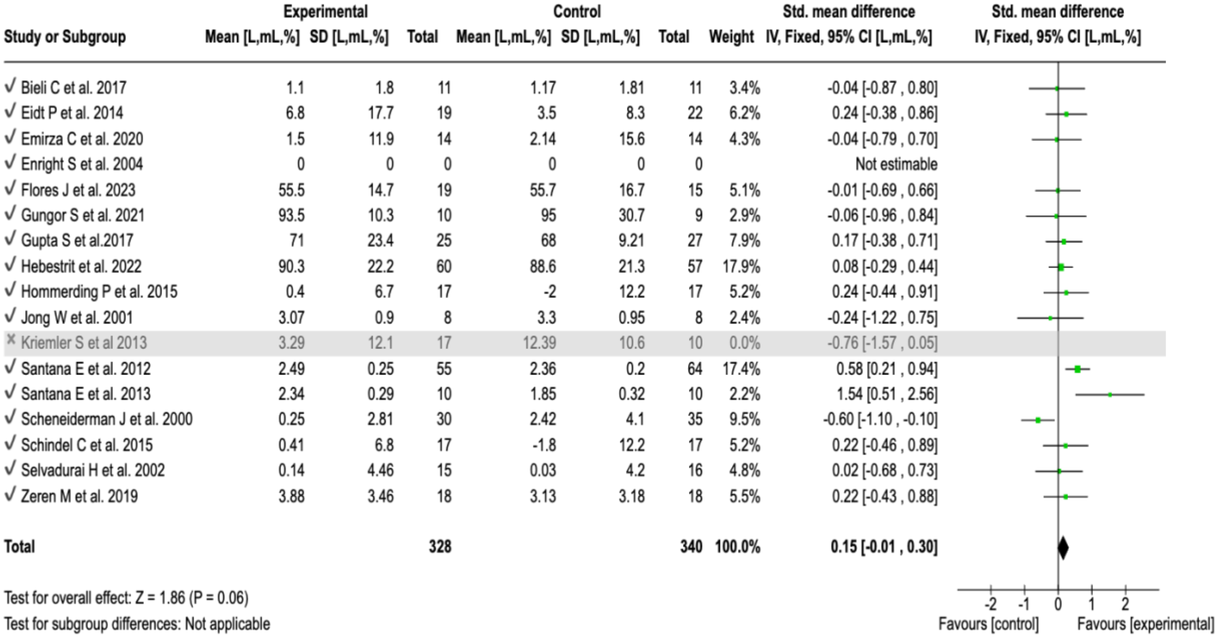

Test for overall effect: Z = 1.86 (P = 0.06)
Test for subgroup differences: Not applicable
Heterogeneity: Chi² = 22.86, df = 14 (P = 0.06); I² = 39%

Figure S8. Sensitivity analysis of the effect of pulmonary rehabilitation and physical training on FVC in patients with cystic fibrosis, excluding the study by Selvadurai H et al[55]

Study or Subgroup	Experimental Mean [L,mL,%]	Experimental SD [L,mL,%]	Experimental Total	Control Mean [L,mL,%]	Control SD [L,mL,%]	Control Total	Weight	Std. mean difference IV, Fixed, 95% CI [L,mL,%]
√ Bieli C et al. 2017	1.1	1.8	11	1.17	1.81	11	3.4%	-0.04 [-0.87 , 0.80]
√ Eidt P et al. 2014	6.8	17.7	19	3.5	8.3	22	6.3%	0.24 [-0.38 , 0.86]
√ Emirza C et al. 2020	1.5	11.9	14	2.14	15.6	14	4.3%	-0.04 [-0.79 , 0.70]
√ Enright S et al. 2004	0	0	0	0	0	0		Not estimable
√ Flores J et al. 2023	55.5	14.7	19	55.7	16.7	15	5.2%	-0.01 [-0.69 , 0.66]
√ Gungor S et al. 2021	93.5	10.3	10	95	30.7	9	2.9%	-0.06 [-0.96 , 0.84]
√ Gupta S et al.2017	71	23.4	25	68	9.21	27	8.0%	0.17 [-0.38 , 0.71]
√ Hebestrit et al. 2022	90.3	22.2	60	88.6	21.3	57	18.2%	0.08 [-0.29 , 0.44]
√ Hommerding P et al. 2015	0.4	6.7	17	-2	12.2	17	5.2%	0.24 [-0.44 , 0.91]
√ Jong W et al. 2001	3.07	0.9	8	3.3	0.95	8	2.5%	-0.24 [-1.22 , 0.75]
√ Kriemler S et al 2013	3.29	12.1	17	12.39	10.6	10	3.6%	-0.76 [-1.57 , 0.05]
√ Santana E et al. 2012	2.49	0.25	55	2.36	0.2	64	17.6%	0.58 [0.21 , 0.94]
√ Santana E et al. 2013	2.34	0.29	10	1.85	0.32	10	2.3%	1.54 [0.51 , 2.56]
√ Scheneiderman J et al. 2000	0.25	2.81	30	2.42	4.1	35	9.6%	-0.60 [-1.10 , -0.10]
√ Schindel C et al. 2015	0.41	6.8	17	-1.8	12.2	17	5.2%	0.22 [-0.46 , 0.89]
× Selvadurai H et al. 2002	0.14	4.46	15	0.03	4.2	16	0.0%	0.02 [-0.68 , 0.73]
√ Zeren M et al. 2019	3.88	3.46	18	3.13	3.18	18	5.6%	0.22 [-0.43 , 0.88]
**Total**			**330**			**334**	**100.0%**	**0.12 [-0.04 , 0.27]**

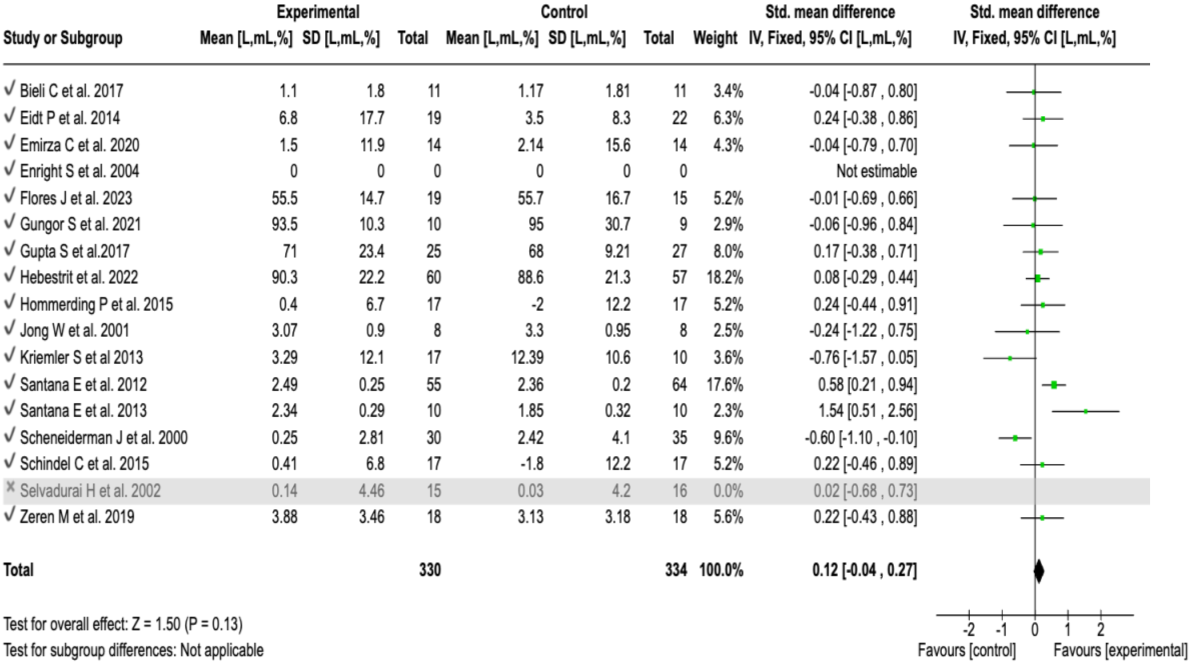

Test for overall effect: Z = 1.50 (P = 0.13)
Test for subgroup differences: Not applicable
Heterogeneity: Chi² = 27.44, df = 14 (P = 0.02); I² = 49%

Figure S9. Sensitivity analysis of the effect of pulmonary rehabilitation and physical training on FEV1/FVC in patients with cystic fibrosis, modified meta-analysis model.

	Experimental			Control				Mean difference
Study or Subgroup	Mean	SD	Total	Mean	SD	Total	Weight	IV, Random, 95% CI
✓ Emirza C et al. 2020	0.14	9.37	14	1.64	10.4	14	18.4%	-1.50 [-8.83 , 5.83]
✓ Flores J et al. 2023	55.5	14.7	19	50.8	15.3	15	9.8%	4.70 [-5.48 , 14.88]
✓ Gungor S et al. 2021	83.3	5.26	10	85.9	5.26	9	41.3%	-2.60 [-7.34 , 2.14]
✓ Hommerding P et al. 2015	0.4	7.7	17	-1	13.2	17	18.8%	1.40 [-5.86 , 8.66]
✓ Sawyer H et al. 1993	87.4	9	10	80.8	12	10	11.7%	6.60 [-2.70 , 15.90]
**Total (Wald[a])**			**70**			**65**	**100.0%**	**0.15 [-3.09 , 3.38]**

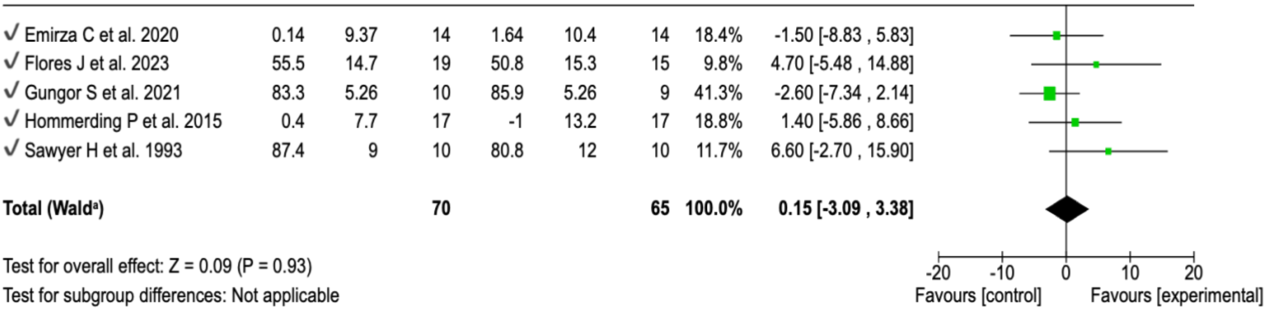

Test for overall effect: Z = 0.09 (P = 0.93)
Test for subgroup differences: Not applicable
Heterogeneity: Tau² (DL[b]) = 0.74; Chi² = 4.21, df = 4 (P = 0.38); I² = 5%

Footnotes
[a]CI calculated by Wald-type method.
[b]Tau² calculated by DerSimonian and Laird method.

Figure S10. Sensitivity analysis of the effect of pulmonary rehabilitation and physical training on RV/TLC in patients with cystic fibrosis, modified meta-analysis model.

	Experimental			Control				Std. mean difference
Study or Subgroup	Mean	SD	Total	Mean	SD	Total	Weight	IV, Random, 95% CI
✔ Hebestrit et al. 2022	0.38	0.13	60	0.37	0.15	57	72.4%	0.07 [-0.29 , 0.43]
✔ Kriemler S et al 2013	1.41	7.4	17	4.59	6.5	10	15.2%	-0.43 [-1.23 , 0.36]
✔ Sawyer H et al. 1993	41	13.1	10	41.6	13.3	10	12.4%	-0.04 [-0.92 , 0.83]
**Total (Wald[a])**			**87**			**77**	**100.0%**	**-0.02 [-0.33 , 0.29]**

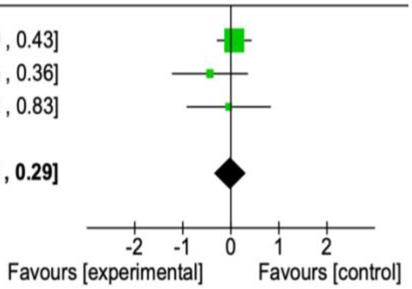

Test for overall effect: Z = 0.13 (P = 0.90)
Test for subgroup differences: Not applicable
Heterogeneity: Tau² (DL[b]) = 0.00; Chi² = 1.30, df = 2 (P = 0.52); I² = 0%

Footnotes
[a]CI calculated by Wald-type method.
[b]Tau² calculated by DerSimonian and Laird method.

Figure S11. Subgroup analysis of the 6-minute walking distance according to age group in patients with cystic fibrosis.

Study or Subgroup	Experimental Mean	Experimental SD	Experimental Total	Control Mean	Control SD	Control Total	Weight	Mean difference IV, Random, 95% CI
**5.3.1 Adult patients**								
Eidt P et al. 2014	7	39.8	19	6.2	37.5	22	20.8%	0.80 [-22.99 , 24.59]
Flores J et al. 2023	57	141	19	23	121.6	15	5.5%	34.00 [-54.35 , 122.35]
Kaltsakar G et al. 2021	583	83	14	564	55	14	11.3%	19.00 [-33.16 , 71.16]
**Subtotal (HKSJ[a])**			**52**			**51**	**37.6%**	**5.64 [-23.68 , 34.95]**
Test for overall effect: T = 0.83, df = 2 (P = 0.50)								
Heterogeneity: Tau² (DL[b]) = 0.00; Chi² = 0.81, df = 2 (P = 0.67); I² = 0%								
**5.3.2 Pediatric patients**								
Corral T et al. 2017	32.12	8.5	20	-8.28	8.3	20	26.4%	40.40 [35.19 , 45.61]
Emirza C et al. 2020	48.54	70.1	14	10.6	61.9	14	12.1%	37.94 [-11.05 , 86.93]
Zeren M et al. 2019	42.8	20.6	18	40.2	26.2	18	23.9%	2.60 [-12.80 , 18.00]
**Subtotal (HKSJ[a])**			**52**			**52**	**62.4%**	**25.51 [-29.20 , 80.22]**
Test for overall effect: T = 2.01, df = 2 (P = 0.18)								
Heterogeneity: Tau² (DL[b]) = 587.62; Chi² = 20.78, df = 2 (P < 0.0001); I² = 90%								
**Total (HKSJ[a])**			**104**			**103**	**100.0%**	**20.05 [-0.15 , 40.25]**

Test for overall effect: T = 2.55, df = 5 (P = 0.05)
Test for subgroup differences: Chi² = 1.09, df = 1 (P = 0.30), I² = 8.3%
Heterogeneity: Tau² (DL[b]) = 520.86; Chi² = 29.46, df = 5 (P < 0.0001); I² = 83%

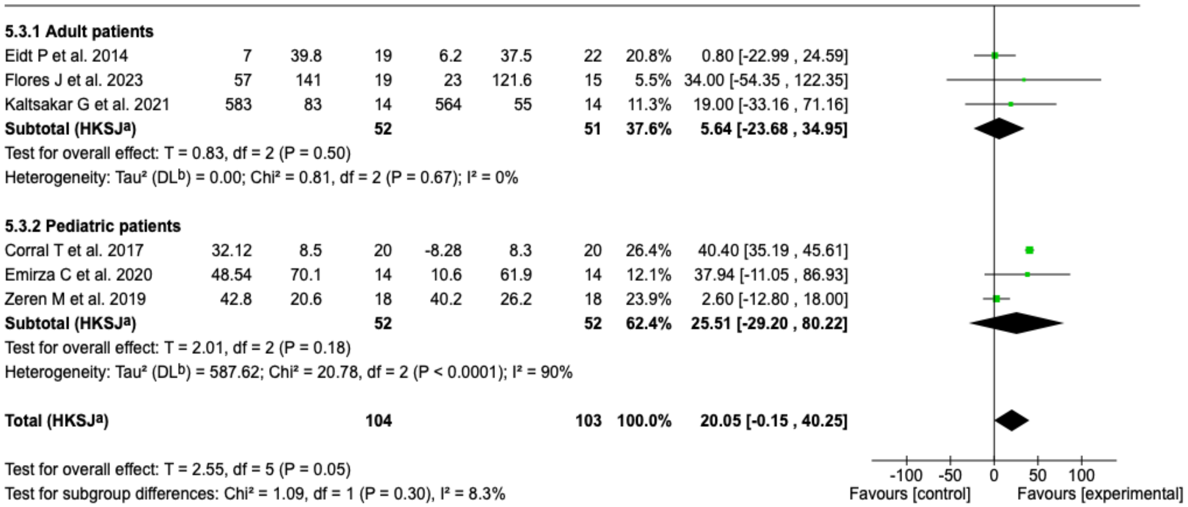

Footnotes
[a]CI calculated by Hartung-Knapp-Sidik-Jonkman method.
[b]Tau² calculated by DerSimonian and Laird method.

Figure S12. Subgroup analysis of the 6-minute walking distance according to treatment duration in patients with cystic fibrosis.

Study or Subgroup	Experimental Mean	Experimental SD	Experimental Total	Control Mean	Control SD	Control Total	Weight	Mean difference IV, Random, 95% CI
**5.2.1 Up to 1 month**								
Flores J et al. 2023	57	141	19	23	121.6	15	5.5%	34.00 [-54.35 , 122.35]
**Subtotal**			**19**			**15**	**5.5%**	**34.00 [-54.35 , 122.35]**
Test for overall effect: Z = 0.75 (P = 0.45)								
Heterogeneity: Not applicable								
**5.2.2 Between 1 and 2 months**								
Corral T et al. 2017	32.12	8.5	20	-8.28	8.3	20	26.4%	40.40 [35.19 , 45.61]
Emirza C et al. 2020	48.54	70.1	14	10.6	61.9	14	12.1%	37.94 [-11.05 , 86.93]
Zeren M et al. 2019	42.8	20.6	18	40.2	26.2	18	23.9%	2.60 [-12.80 , 18.00]
**Subtotal (HKSJ[a])**			**52**			**52**	**62.4%**	**25.51 [-29.20 , 80.22]**
Test for overall effect: T = 2.01, df = 2 (P = 0.18)								
Heterogeneity: Tau² (DL[b]) = 587.62; Chi² = 20.78, df = 2 (P < 0.0001); I² = 90%								
**5.2.3 At least 3 months**								
Eidt P et al. 2014	7	39.8	19	6.2	37.5	22	20.8%	0.80 [-22.99 , 24.59]
Kaltsakar G et al. 2021	583	83	14	564	55	14	11.3%	19.00 [-33.16 , 71.16]
**Subtotal (HKSJ[a])**			**33**			**36**	**32.2%**	**3.93 [-83.37 , 91.24]**
Test for overall effect: T = 0.57, df = 1 (P = 0.67)								
Heterogeneity: Tau² (DL[b]) = 0.00; Chi² = 0.39, df = 1 (P = 0.53); I² = 0%								
**Total (HKSJ[a])**			**104**			**103**	**100.0%**	**20.05 [-0.15 , 40.25]**

Test for overall effect: T = 2.55, df = 5 (P = 0.05)
Test for subgroup differences: Chi² = 1.51, df = 2 (P = 0.47), I² = 0%
Heterogeneity: Tau² (DL[b]) = 520.86; Chi² = 29.46, df = 5 (P < 0.0001); I² = 83%

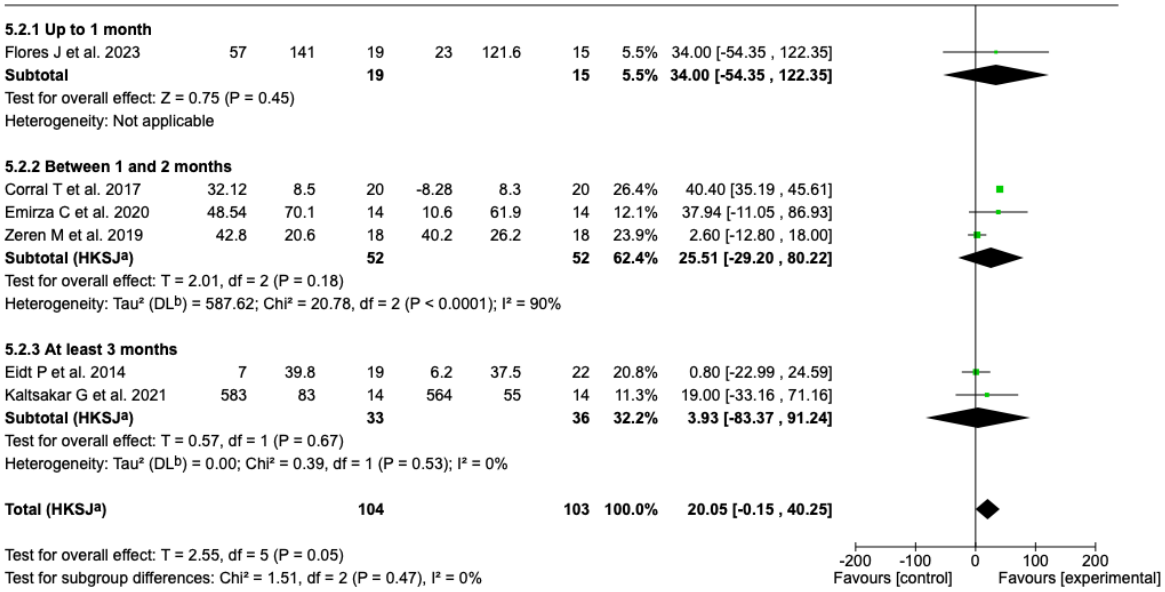

Footnotes
[a]CI calculated by Hartung-Knapp-Sidik-Jonkman method.
[b]Tau² calculated by DerSimonian and Laird method.

Figure S13. Sensitivity analysis of the effect of pulmonary rehabilitation and physical training on 6MWD in patients with cystic fibrosis, modified meta-analysis model.

	Experimental			Control				Mean difference
Study or Subgroup	Mean	SD	Total	Mean	SD	Total	Weight	IV, Fixed, 95% CI
√ Corral T et al. 2017	32.12	8.5	20	-8.28	8.3	20	84.2%	40.40 [35.19 , 45.61]
√ Eidt P et al. 2014	7	39.8	19	6.2	37.5	22	4.0%	0.80 [-22.99 , 24.59]
√ Emirza C et al. 2020	48.54	70.1	14	10.6	61.9	14	1.0%	37.94 [-11.05 , 86.93]
√ Flores J et al. 2023	57	141	19	23	121.6	15	0.3%	34.00 [-54.35 , 122.35]
√ Kaltsakar G et al. 2021	583	83	14	564	55	14	0.8%	19.00 [-33.16 , 71.16]
√ Zeren M et al. 2019	42.8	20.6	18	40.2	26.2	18	9.6%	2.60 [-12.80 , 18.00]
**Total**			**104**			**103**	**100.0%**	**34.94 [30.16 , 39.72]**

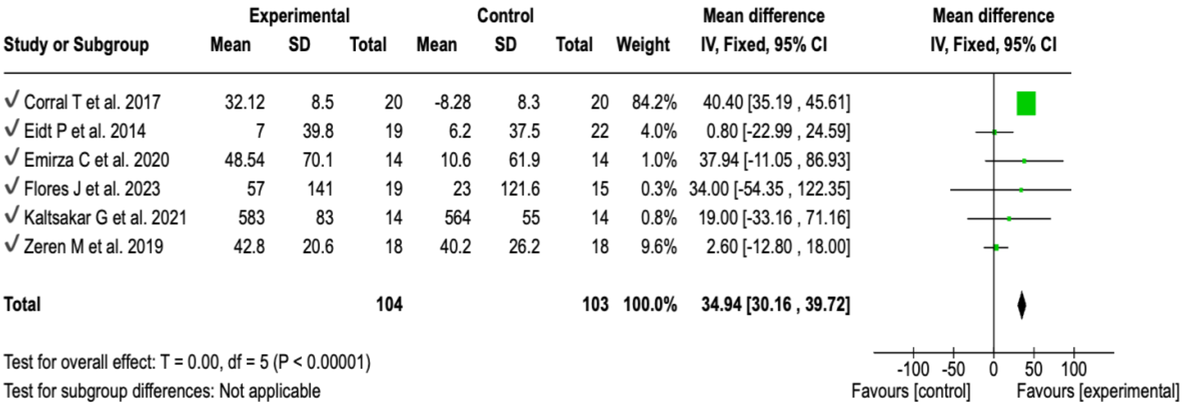

Test for overall effect: T = 0.00, df = 5 (P < 0.00001)
Test for subgroup differences: Not applicable
Heterogeneity: Chi² = 29.46, df = 5 (P < 0.0001); I² = 83%

Figure S14. Subgroup analysis of the VO2 max. according to age group in patients with cystic fibrosis.

	Experimental			Control				Mean difference
Study or Subgroup	Mean	SD	Total	Mean	SD	Total	Weight	IV, Random, 95% CI
**6.3.1 Adult patients**								
Hebestrit et al. 2022	78.5	18.8	60	73.8	15.9	57	8.0%	4.70 [-1.60 , 11.00]
Jong W et al. 2001	29	6	8	30	11	8	5.6%	-1.00 [-9.68 , 7.68]
Kaltsakar G et al. 2021	26.9	11	14	25.7	9.5	14	6.5%	1.20 [-6.41 , 8.81]
Kriemler S et al 2013	4.5	13.8	17	7.36	12.9	10	4.4%	-2.86 [-13.20 , 7.48]
**Subtotal (HKSJ[a])**			**99**			**89**	**24.5%**	**1.52 [-3.67 , 6.72]**
Test for overall effect: T = 0.93, df = 3 (P = 0.42)								
Heterogeneity: Tau² (DL[b]) = 0.00; Chi² = 2.00, df = 3 (P = 0.57); I² = 0%								
**6.3.2 Pediatric patients**								
Gupta S et al.2017	32.5	8	25	25.5	6	27	11.4%	7.00 [3.13 , 10.87]
Hommerding P et al. 2015	1.1	4.6	17	2.3	11.9	17	8.3%	-1.20 [-7.26 , 4.86]
Klijin P et al. 2004	88	106	11	48	63	9	0.1%	40.00 [-34.95 , 114.95]
Santana E et al. 2012	3.9	1.1	55	-2.2	1.382	64	15.3%	6.10 [5.65 , 6.55]
Santana E et al. 2013	38	1.3	10	35.6	1.5	10	14.9%	2.40 [1.17 , 3.63]
Scheneiderman J et al. 2000	1.8	2.21	30	1.85	2.51	35	14.9%	-0.05 [-1.20 , 1.10]
Selvadurai H et al. 2002	0.25	6.5	15	-3.87	6	16	10.6%	4.12 [-0.29 , 8.53]
**Subtotal (HKSJ[a])**			**163**			**178**	**75.5%**	**3.27 [0.03 , 6.50]**
Test for overall effect: T = 2.47, df = 6 (P = 0.05)								
Heterogeneity: Tau² (DL[b]) = 11.49; Chi² = 120.92, df = 6 (P < 0.00001); I² = 95%								
**Total (HKSJ[a])**			**262**			**267**	**100.0%**	**2.74 [0.43 , 5.04]**

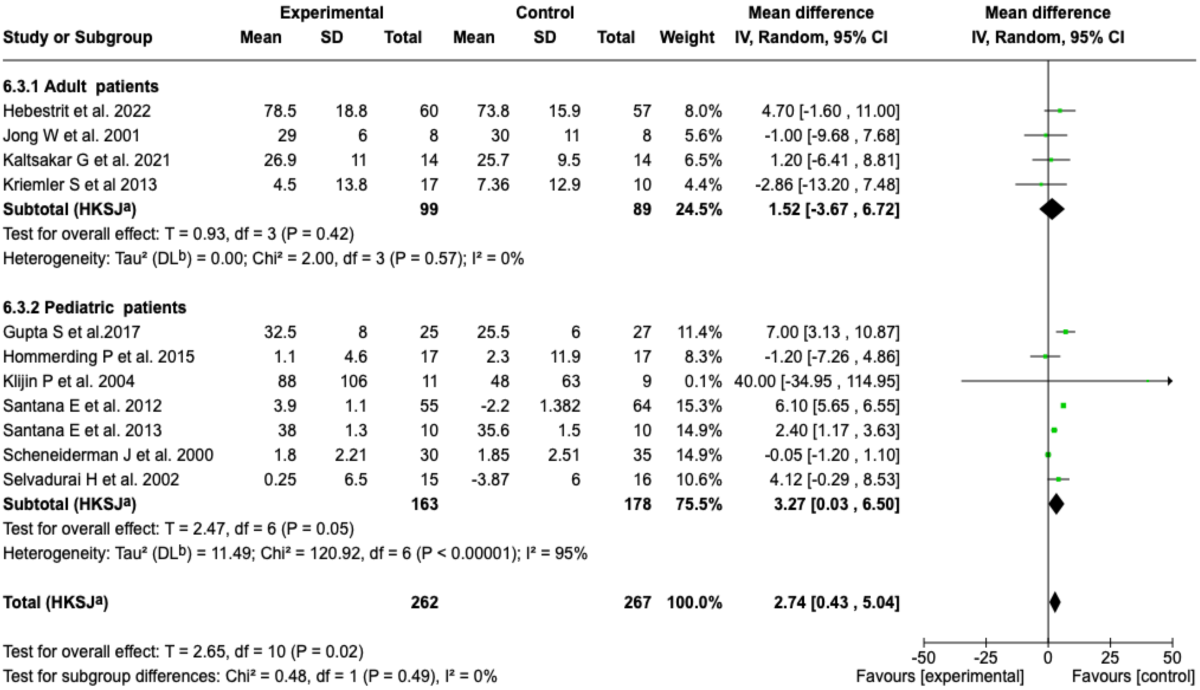

Test for overall effect: T = 2.65, df = 10 (P = 0.02)
Test for subgroup differences: Chi² = 0.48, df = 1 (P = 0.49), I² = 0%
Heterogeneity: Tau² (DL[b]) = 11.14; Chi² = 125.88, df = 10 (P < 0.00001); I² = 92%

Footnotes
[a]CI calculated by Hartung-Knapp-Sidik-Jonkman method.
[b]Tau² calculated by DerSimonian and Laird method.

Figure S15. Subgroup analysis of VO2 max. according to treatment duration in patients with cystic fibrosis.

Study or Subgroup	Experimental Mean	Experimental SD	Experimental Total	Control Mean	Control SD	Control Total	Weight	Mean difference IV, Random, 95% CI
**6.2.1 Up to 1 month**								
Selvadurai H et al. 2002	0.25	6.5	15	-3.87	6	16	10.6%	4.12 [-0.29 , 8.53]
**Subtotal**			**15**			**16**	**10.6%**	**4.12 [-0.29 , 8.53]**
Test for overall effect: Z = 1.83 (P = 0.07)								
Heterogeneity: Not applicable								
**6.2.2 Between 1 and 2 months**								
Jong W et al. 2001	29	6	8	30	11	8	5.6%	-1.00 [-9.68 , 7.68]
Santana E et al. 2013	38	1.3	10	35.6	1.5	10	14.9%	2.40 [1.17 , 3.63]
**Subtotal (HKSJ[a])**			**18**			**18**	**20.4%**	**2.33 [-3.67 , 8.33]**
Test for overall effect: T = 4.94, df = 1 (P = 0.13)								
Heterogeneity: Tau² (DL[b]) = 0.00; Chi² = 0.58, df = 1 (P = 0.45); I² = 0%								
**6.2.3 At least 3 months**								
Gupta S et al.2017	32.5	8	25	25.5	6	27	11.4%	7.00 [3.13 , 10.87]
Hebestrit et al. 2022	78.5	18.8	60	73.8	15.9	57	8.0%	4.70 [-1.60 , 11.00]
Hommerding P et al. 2015	1.1	4.6	17	2.3	11.9	17	8.3%	-1.20 [-7.26 , 4.86]
Kaltsakar G et al. 2021	26.9	11	14	25.7	9.5	14	6.5%	1.20 [-6.41 , 8.81]
Klijin P et al. 2004	88	106	11	48	63	9	0.1%	40.00 [-34.95 , 114.95]
Kriemler S et al 2013	4.5	13.8	17	7.36	12.9	10	4.4%	-2.86 [-13.20 , 7.48]
Santana E et al. 2012	3.9	1.1	55	-2.2	1.382	64	15.3%	6.10 [5.65 , 6.55]
Scheneiderman J et al. 2000	1.8	2.21	30	1.85	2.51	35	14.9%	-0.05 [-1.20 , 1.10]
**Subtotal (HKSJ[a])**			**229**			**233**	**69.0%**	**2.80 [-0.65 , 6.26]**
Test for overall effect: T = 1.92, df = 7 (P = 0.10)								
Heterogeneity: Tau² (DL[b]) = 16.26; Chi² = 105.31, df = 7 (P < 0.00001); I² = 93%								
**Total (HKSJ[a])**			**262**			**267**	**100.0%**	**2.74 [0.43 , 5.04]**

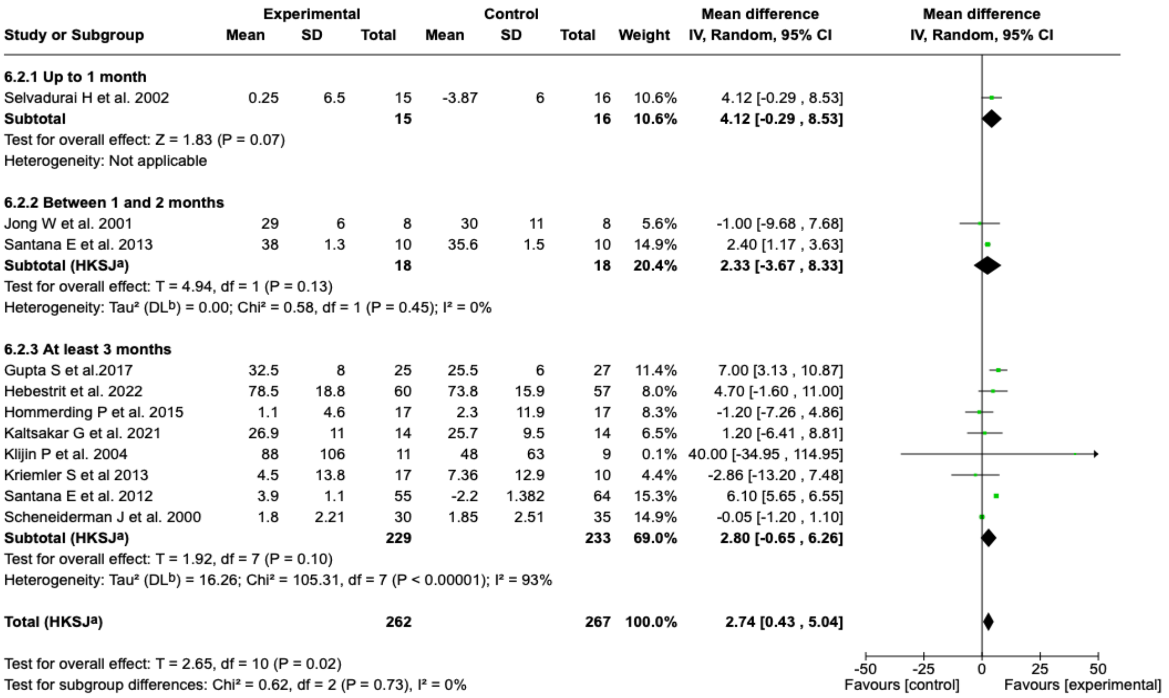

Test for overall effect: T = 2.65, df = 10 (P = 0.02)
Test for subgroup differences: Chi² = 0.62, df = 2 (P = 0.73), I² = 0%
Heterogeneity: Tau² (DL[b]) = 11.14; Chi² = 125.88, df = 10 (P < 0.00001); I² = 92%

Footnotes
[a]CI calculated by Hartung-Knapp-Sidik-Jonkman method.
[b]Tau² calculated by DerSimonian and Laird method.

Figure S16. Sensitivity analysis of the effect of pulmonary rehabilitation and physical training on VO2 max. in patients with cystic fibrosis, excluding the study by Kriemler S et al. [48]

Study or Subgroup	Experimental Mean	Experimental SD	Experimental Total	Control Mean	Control SD	Control Total	Weight	Mean difference IV, Random, 95% CI
✓ Gupta S et al.2017	32.5	8	25	25.5	6	27	11.9%	7.00 [3.13 , 10.87]
✓ Hebestrit et al. 2022	78.5	18.8	60	73.8	15.9	57	8.3%	4.70 [-1.60 , 11.00]
✓ Hommerding P et al. 2015	1.1	4.6	17	2.3	11.9	17	8.6%	-1.20 [-7.26 , 4.86]
✓ Jong W et al. 2001	29	6	8	30	11	8	5.8%	-1.00 [-9.68 , 7.68]
✓ Kaltsakar G et al. 2021	26.9	11	14	25.7	9.5	14	6.8%	1.20 [-6.41 , 8.81]
✓ Klijin P et al. 2004	88	106	11	48	63	9	0.1%	40.00 [-34.95 , 114.95]
× Kriemler S et al 2013	4.5	13.8	17	7.36	12.9	10	0.0%	-2.86 [-13.20 , 7.48]
✓ Santana E et al. 2012	3.9	1.1	55	-2.2	1.382	64	16.0%	6.10 [5.65 , 6.55]
✓ Santana E et al. 2013	38	1.3	10	35.6	1.5	10	15.6%	2.40 [1.17 , 3.63]
✓ Scheneiderman J et al. 2000	1.8	2.21	30	1.85	2.51	35	15.6%	-0.05 [-1.20 , 1.10]
✓ Selvadurai H et al. 2002	0.25	6.5	15	-3.87	6	16	11.1%	4.12 [-0.29 , 8.53]
**Total (HKSJ[a])**			**245**			**257**	**100.0%**	**3.00 [0.65 , 5.34]**

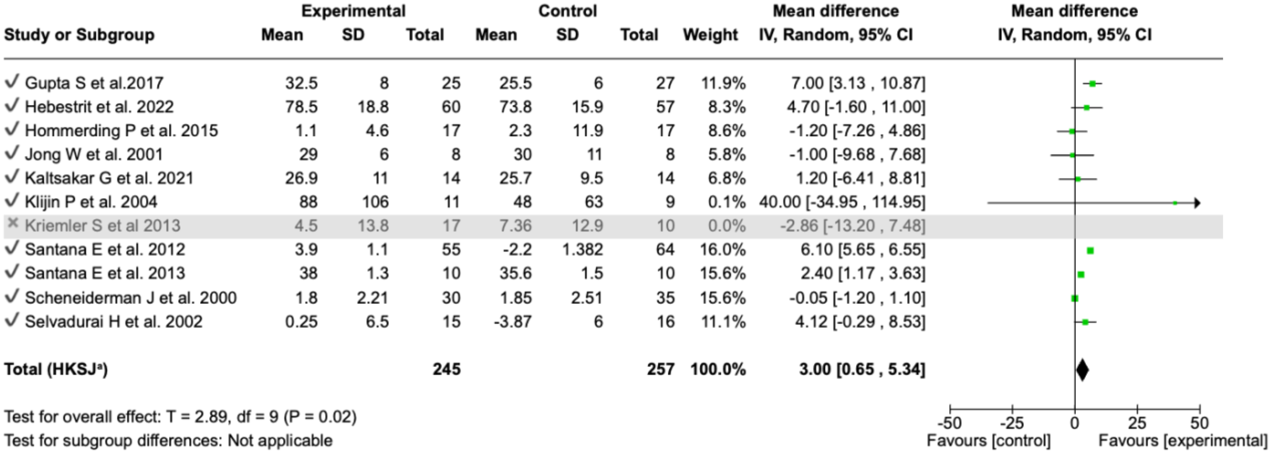

Test for overall effect: T = 2.89, df = 9 (P = 0.02)
Test for subgroup differences: Not applicable
Heterogeneity: Tau² (DL[b]) = 11.09; Chi² = 123.69, df = 9 (P < 0.00001); I² = 93%

Figure S17. Sensitivity analysis of the effect of pulmonary rehabilitation and physical training on VO2 max. in patients with cystic fibrosis, excluding the study by Selvadurai H et al [55]

Study or Subgroup	Experimental Mean	Experimental SD	Experimental Total	Control Mean	Control SD	Control Total	Weight	Mean difference IV, Random, 95% CI
✓ Gupta S et al.2017	32.5	8	25	25.5	6	27	12.8%	7.00 [3.13 , 10.87]
✓ Hebestrit et al. 2022	78.5	18.8	60	73.8	15.9	57	9.0%	4.70 [-1.60 , 11.00]
✓ Hommerding P et al. 2015	1.1	4.6	17	2.3	11.9	17	9.3%	-1.20 [-7.26 , 4.86]
✓ Jong W et al. 2001	29	6	8	30	11	8	6.3%	-1.00 [-9.68 , 7.68]
✓ Kaltsakar G et al. 2021	26.9	11	14	25.7	9.5	14	7.4%	1.20 [-6.41 , 8.81]
✓ Klijin P et al. 2004	88	106	11	48	63	9	0.1%	40.00 [-34.95 , 114.95]
✓ Kriemler S et al 2013	4.5	13.8	17	7.36	12.9	10	5.0%	-2.86 [-13.20 , 7.48]
✓ Santana E et al. 2012	3.9	1.1	55	-2.2	1.382	64	17.0%	6.10 [5.65 , 6.55]
✓ Santana E et al. 2013	38	1.3	10	35.6	1.5	10	16.5%	2.40 [1.17 , 3.63]
✓ Scheneiderman J et al. 2000	1.8	2.21	30	1.85	2.51	35	16.6%	-0.05 [-1.20 , 1.10]
× Selvadurai H et al. 2002	0.25	6.5	15	-3.87	6	16	0.0%	4.12 [-0.29 , 8.53]
**Total (HKSJ[a])**			**247**			**251**	**100.0%**	**2.56 [-0.03 , 5.15]**

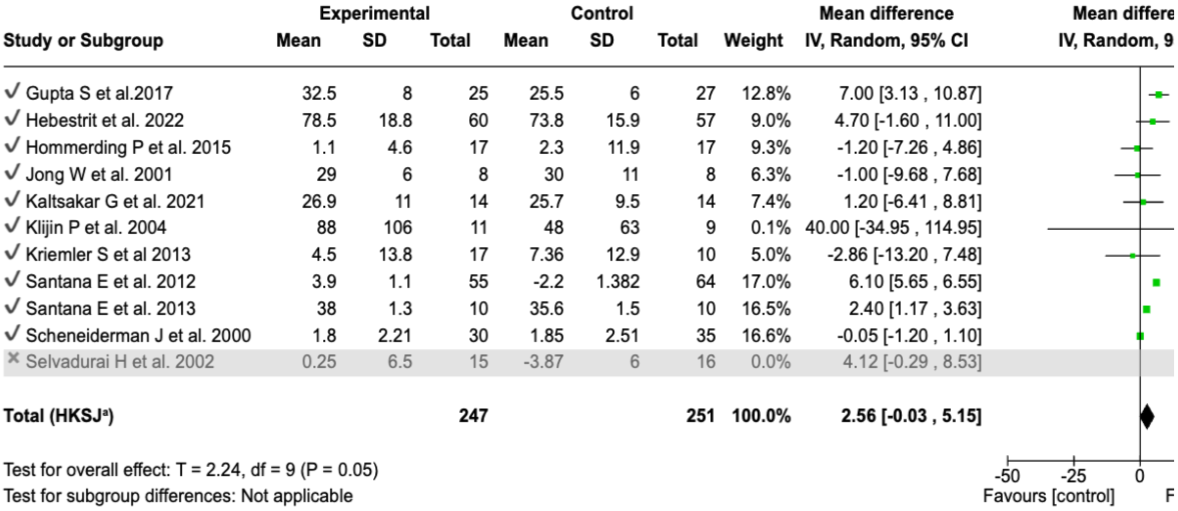

Test for overall effect: T = 2.24, df = 9 (P = 0.05)
Test for subgroup differences: Not applicable
Heterogeneity: Tau^2 (DL[b]) = 11.57; Chi^2 = 125.74, df = 9 (P < 0.00001); I^2 = 93%

Figure S18. Sensitivity analysis of the effect of pulmonary rehabilitation and physical training [illegible] W max in patients with cystic fibrosis, modified meta-analysis model.

Study or Subgroup	Experimental Mean	Experimental SD	Experimental Total	Control Mean	Control SD	Control Total	Weight	Std. mean difference IV, Random, 95% CI
✓ Hebestrit et al. 2022	90.9	16.8	60	87.9	18.4	57	29.1%	0.17 [-0.19 , 0.53]
✓ Jong W et al. 2001	106	44	8	126	48	8	6.6%	-0.41 [-1.40 , 0.58]
✓ Kaltsakar G et al. 2021	108	60	14	109	59	14	11.0%	-0.02 [-0.76 , 0.72]
✓ Klijin P et al. 2004	11	14	11	-2	5	9	7.0%	1.14 [0.17 , 2.10]
✓ Kriemler S et al 2013	-0.1	0.6	17	-0.23	0.6	10	10.0%	0.21 [-0.57 , 0.99]
✓ Moorcroft et al. 2004	142	51	30	154	41	18	15.8%	-0.25 [-0.83 , 0.34]
✓ Scheneiderman J et al. 2000	1.68	5.16	30	2.5	6.08	35	20.5%	-0.14 [-0.63 , 0.35]
**Total (Wald[a])**			**170**			**151**	**100.0%**	**0.05 [-0.22 , 0.32]**

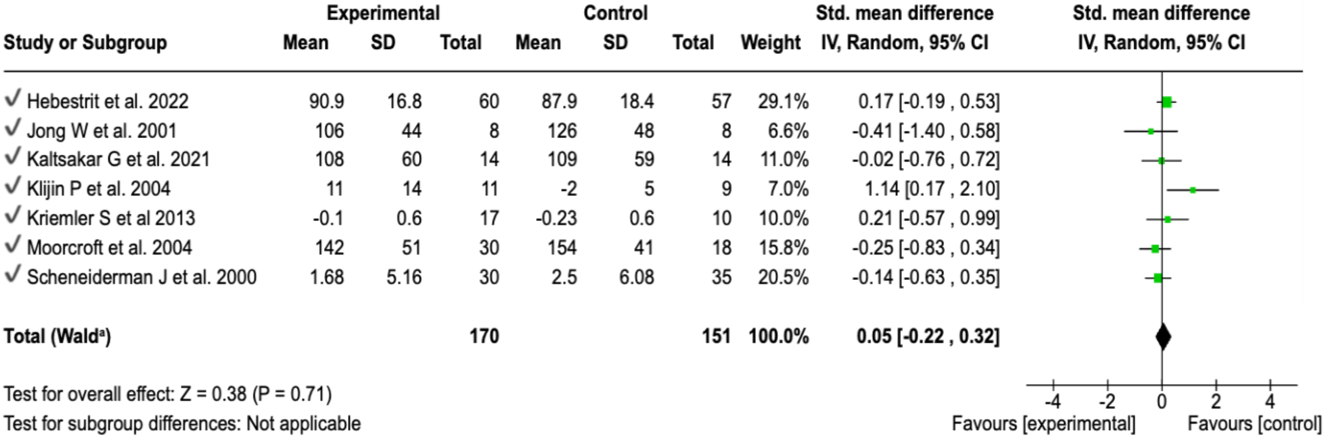

Test for overall effect: Z = 0.38 (P = 0.71)
Test for subgroup differences: Not applicable
Heterogeneity: Tau^2 (DL[b]) = 0.03; Chi^2 = 7.90, df = 6 (P = 0.25); I^2 = 24%

Figure S19. Sensitivity analysis of the effect of pulmonary rehabilitation and physical training on W max. in patients with cystic fibrosis, excluding the study by Kriemler S et al. [48]

Study or Subgroup	Experimental Mean	Experimental SD	Experimental Total	Control Mean	Control SD	Control Total	Weight	Std. mean difference IV, Fixed, 95% CI
✓ Hebestrit et al. 2022	90.9	16.8	60	87.9	18.4	57	40.8%	0.17 [-0.19 , 0.53]
✓ Jong W et al. 2001	106	44	8	126	48	8	5.4%	-0.41 [-1.40 , 0.58]
✓ Kaltsakar G et al. 2021	108	60	14	109	59	14	9.8%	-0.02 [-0.76 , 0.72]
✓ Klijin P et al. 2004	11	14	11	-2	5	9	5.8%	1.14 [0.17 , 2.10]
× Kriemler S et al 2013	-0.1	0.6	17	-0.23	0.6	10	0.0%	0.21 [-0.57 , 0.99]
✓ Moorcroft et al. 2004	142	51	30	154	41	18	15.6%	-0.25 [-0.83 , 0.34]
✓ Scheneiderman J et al. 2000	1.68	5.16	30	2.5	6.08	35	22.6%	-0.14 [-0.63 , 0.35]
**Total**			**153**			**141**	**100.0%**	**0.04 [-0.19 , 0.27]**

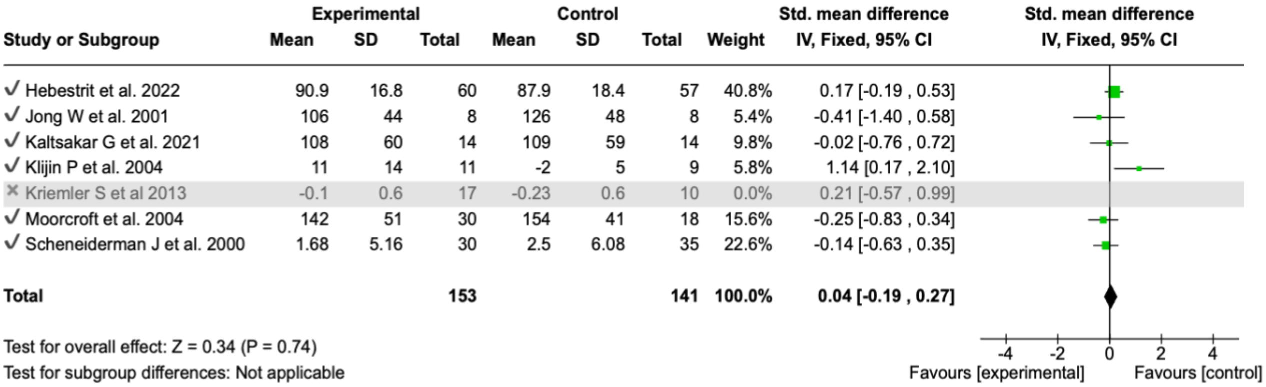

Test for overall effect: Z = 0.34 (P = 0.74)
Test for subgroup differences: Not applicable
Heterogeneity: Chi^2 = 7.73, df = 5 (P = 0.17); I^2 = 35%
