# Supplementary material for: Physical Training and Pulmonary Rehabilitation in Patients with Cystic Fibrosis: A Systematic Review and Meta-Analysis of Clinical Trials
Source: Healthcare (Basel). 2025 Aug 15;13(16):2017. doi: 10.3390/healthcare13162017 (PMC12385196; doi:10.3390/healthcare13162017)
Supplement: Supplementary file 1 [file healthcare-13-02017-s001.zip › Table S1.pdf]

Table S1. Summary of Meta-Analysis Findings: Effects of Physical Training and Pulmonary Rehabilitation on Clinical Outcomes in Patients with Cystic Fibrosis

Outcome	No. of Studies	Participant	Effect Size (MD or SMD)	CI 95%	Direction of Effect	Certainty of the Evidence
Pulmonary function (FEV1)	19	754	0.05	-0.09 to 0.20	✖	Low
Pulmonary function (FVC)	17	695	0.11	-0.04 to 0.27	✖	Low
Pulmonary function (FEV1/FVC)	5	135	0.04	-3.07 to 3.15	✖	Low
Pulmonary function (RV/TLC)	3	164	-0.02	-0.33 to 0.29	✖	Low
Exercise capacity (6MWT)	6	207	20.05	-0.15 to 40.25	✖	Very low
Exercise capacity (VO2 max)	11	529	2.74	0.43 to 5.04	✔	Low
Exercise capacity (Wmax)	7	321	0.05	-0.17 to 0.28	✖	Low

✔ In favor of the intervention ✖ No effect
