# Supplementary material for: Physical Training and Pulmonary Rehabilitation in Patients with Cystic Fibrosis: A Systematic Review and Meta-Analysis of Clinical Trials
Source: Healthcare (Basel). 2025 Aug 15;13(16):2017. doi: 10.3390/healthcare13162017 (PMC12385196; doi:10.3390/healthcare13162017)
Supplement: Supplementary file 1 [file healthcare-13-02017-s001.zip › Table S2.pdf]

Author(s): Angie Melissa Hinestroza Mancilla, Lina Manuela Pérez Ordoñez, Naudy Yulisa Ararat Carabalí, Saray Ríos Murillo, Freiser Eceomo Cruz Mosquera, Yamil Liscano.
Question: Pulmonary rehabilitation or physical training compared to standard care for cystic fibrosis
Setting:
Bibliography: 1. Knobloch, K.; Yoon, U.; Vogt, P.M. Preferred Reporting Items for Systematic Reviews and Meta-Analyses (PRISMA) Statement and Publication Bias. Journal of Cranio-Maxillofacial Surgery 2011, 39, 91–92, doi:10.1016/j.jcms.2010.11.001.2. Higgins, J.P.T.; Altman, D.G.; Gotzsche, P.C.; Juni, P.; Moher, D.; Oxman, A.D.; Savovic, J.; Schulz, K.F.; Weeks, L.; Sterne, J.A.C.; et al. The Cochrane Collaboration's Tool for Assessing Risk of Bias in Randomised Trials. BMJ 2011, 343, d5928–d5928, doi:10.1136/bmj.d5928.3. Flores, J.; Ziegler, B.; Silvello, D.; Dalcin, P.T.R. Effects of an Early Rehabilitation Program for Adult Cystic Fibrosis Patients during Hospitalization: A Randomized Clinical Trial. Braz J Med Biol Res 2023, 56, e12752, doi:10.1590/1414-431x2023e12752.4. Hebestreit, H.; Kriemler, S.; Schindler, C.; Stein, L.; Karila, C.; Urquhart, D.S.; Orenstein, D.M.; Lands, L.C.; Schaeff, J.; Eber, E.; et al. Effects of a Partially Supervised Conditioning Program in Cystic Fibrosis: An International Multicenter, Randomized Controlled Trial (ACTIVATE-CF). Am J Respir Crit Care Med 2022, 205, 330–339, doi:10.1164/rccm.202106-1419OC.5. Kaltsakas, G.; Chynkiamis, N.; Anastasopoulos, N.; Zeliou, P.; Karapatoucha, V.; Kotsifas, K.; Diamantea, F.; Inglezos, I.; Koulouris, N.G.; Vogiatzis, I. Interval versus Constant-Load Exercise Training in Adults with Cystic Fibrosis. Respiratory Physiology & Neurobiology 2021, 288, 103643, doi:10.1016/j.resp.2021.103643.6. Güngör, S. The Clinical Effects of Combining Postural Exercises with Chest Physiotherapy in Cystic Fibrosis: A Single-Blind, Randomized-Controlled Trial. Turk J Phys Med Rehab 2021, 67, 91–98, doi:10.5606/tftrd.2021.5214.7. Emirza, C.; Aslan, G.K.; Kilinc, A.A.; Cokugras, H. Effect of Expiratory Muscle Training on Peak Cough Flow in Children and Adolescents with Cystic Fibrosis: A Randomized Controlled Trial. Pediatric Pulmonology 2021, 56, 939–947, doi:10.1002/ppul.25259.8. Gupta, S.; Mukherjee, A.; Lodha, R.; Kabra, M.; Deepak, K.K.; Khadgawat, R.; Talwar, A.; Kabra, S.K. Effects of Exercise Intervention Program on Bone Mineral Accretion in Children and Adolescents with Cystic Fibrosis: A Randomized Controlled Trial. Indian J Pediatr 2019, 86, 987–994, doi:10.1007/s12098-019-03019-x.9. Zeren, M.; Cakir, E.; Gurses, H.N. Effects of Inspiratory Muscle Training on Postural Stability, Pulmonary Function and Functional Capacity in Children with Cystic Fibrosis: A Randomised Controlled Trial. Respiratory Medicine 2019, 148, 24–30, doi:10.1016/j.rmed.2019.01.013.10. Bieli, C.; Summermatter, S.; Boutellier, U.; Moeller, A. Respiratory Muscle Training Improves Respiratory Muscle Endurance but Not Exercise Tolerance in Children with Cystic Fibrosis. Pediatric Pulmonology 2017, 52, 331–336, doi:10.1002/ppul.23647.11. Del Corral, T.; Cebrià I Iranzo, M.À.; López-de-Uralde-Villanueva, I.; Martínez-Alejos, R.; Blanco, I.; Vilaró, J. Effectiveness of a Home-Based Active Video Game Programme in Young Cystic Fibrosis Patients. Respiration 2018, 95, 87–97, doi:10.1159/000481264.12. Schindel, C.S.; Hommerding, P.X.; Melo, D.A.S.; Baptista, R.R.; Marostica, P.J.C.; Donadio, M.V.F. Physical Exercise Recommendations Improve Postural Changes Found in Children and Adolescents with Cystic Fibrosis: A Randomized Controlled Trial. The Journal of Pediatrics 2015, 166, 710-716.e2, doi:10.1016/j.jpeds.2014.12.001.13. Hommerding, P.X.; Baptista, R.R.; Makarewicz, G.T.; Schindel, C.S.; Donadio, M.V.; Pinto, L.A.; Marostica, P.J. Effects of an Educational Intervention of Physical Activity for Children and Adolescents With Cystic Fibrosis: A Randomized Controlled Trial. Respiratory Care 2015, 60, 81–87, doi:10.4187/respcare.02578.14. Eidt, P.M.; Flores, J.; Ziegler, B.; Casarotto, F.; Jaques, P.; Barreto, S.S.M.; Dalcin, P.D.T.R. Exercise Programme in Patients with Cystic Fibrosis: A Randomized Controlled Trial. Respiratory Medicine 2014, 108, 1134–1140, doi:10.1016/j.rmed.2014.04.022.15. Kriemler, S.; Kieser, S.; Junge, S.; Ballmann, M.; Hebestreit, A.; Schindler, C.; Stüssi, C.; Hebestreit, H. Effect of Supervised Training on FEV1 in Cystic Fibrosis: A Randomised Controlled Trial. Journal of Cystic Fibrosis 2013, 12, 714–720, doi:10.1016/j.jcf.2013.03.003.16. Santana-Sosa, E.; Gonzalez-Saiz, L.; Groeneveld, I.F.; Villa-Asensi, J.R.; Barrio Gómez De Aguero, M.I.; Fleck, S.J.; López-Mojares, L.M.; Pérez, M.; Lucia, A. Benefits of Combining Inspiratory Muscle with 'Whole Muscle' Training in Children with Cystic Fibrosis: A Randomised Controlled Trial. Br J Sports Med 2014, 48, 1513–1517, doi:10.1136/bjsports-2012-091892.17. Santana, E.; Groeneveld, I.F.; Gonzalez-Saiz, L.; López-Mojares, L.M.; Villa-Asensi, J.R.; Gonzalez, M.I.B.; Fleck, S.J.; Pérez, M.; Lucia, A. Intrahospital Weight and Aerobic Training in Children with Cystic Fibrosis: A Randomized Controlled Trial. Medicine & Science in Sports & Exercise 2012, 44, 2–11, doi:10.1249/MSS.0b013e318228c302.18. Sandsund, C.A.; Roughton, M.; Hodson, M.E.; Pryor, J.A. Musculoskeletal Techniques for Clinically Stable Adults with Cystic Fibrosis: A Preliminary Randomised Controlled Trial. Physiotherapy 2011, 97, 209–217, doi:10.1016/j.physio.2010.08.016.19. Klijn, P.H.C.; Oudshoorn, A.; Van Der Ent, C.K.; Van Der Net, J.; Kimpen, J.L.; Helders, P.J.M. Effects of Anaerobic Training in Children With Cystic Fibrosis. Chest 2004, 125, 1299–1305, doi:10.1378/chest.125.4.1299.20. Enright, S.; Chatham, K.; Ionescu, A.A.; Unnithan, V.B.; Shale, D.J. Inspiratory Muscle Training Improves Lung Function and Exercise Capacity in Adults With Cystic Fibrosis. Chest 2004, 126, 405–411, doi:10.1378/chest.126.2.405.21. Moorcroft, A.J. Individualised Unsupervised Exercise Training in Adults with Cystic Fibrosis: A 1 Year Randomised Controlled Trial. Thorax 2004, 59, 1074–1080, doi:10.1136/thx.2003.015313.22. Selvadurai, H.C.; Blimkie, C.J.; Meyers, N.; Mellis, C.M.; Cooper, P.J.; Van Asperen, P.P. Randomized Controlled Study of In-hospital Exercise Training Programs in Children with Cystic Fibrosis. Pediatric Pulmonology 2002, 33, 194–200, doi:10.1002/ppul.10015.23. De Jong, W.; Van Aalderen, W.M.C.; Kraan, J.; Koëter, G.H.; Van Der Schans, C.P. Inspiratory Muscle Training in Patients with Cystic Fibrosis. Respiratory Medicine 2001, 95, 31–36, doi:10.1053/rmed.2000.0966.24. Schneiderman-Walker, J.; Pollock, S.L.; Corey, M.; Wilkes, D.D.; Canny, G.J.; Pedder, L.; Reisman, J.J. A Randomized Controlled Trial of a 3-Year Home Exercise Program in Cystic Fibrosis. The Journal of Pediatrics 2000, 136, 304–310, doi:10.1067/mpd.2000.103408.25. Sawyer, E.H.; Clanton, T.L. Improved Pulmonary Function and Exercise Tolerance With Inspiratory Muscle Conditioning in Children With Cystic Fibrosis. Chest 1993, 104, 1490–1497, doi:10.1378/chest.104.5.1490.26. Schilder, A.G.M.; Chonmaitree, T.; Cripps, A.W.; Rosenfeld, R.M.; Casselbrant, M.L.; Haggard, M.P.; Venekamp, R.P. Otitis Media. Nat Rev Dis Primers 2016, 2, 16063, doi:10.1038/nrdp.2016.63.

Certainty assessment							№ of patients		Effect		Certainty	Importance
№ of studies	Study design	Risk of bias	Inconsistency	Indirectness	Imprecision	Other considerations	pulmonary rehabilitation or physical training	standard care	Relative (95% CI)	Absolute (95% CI)		
**Lung function (FEV1)**												
19	randomised trials	serious[a]	not serious	not serious	serious[b]	none	374	380	-	**0** (0 to 0)	⊕⊕◯◯ Low[a,b]	
**lung function (FVC)**												
17	randomised trials	serious[a]	not serious	not serious	serious[b]	none	345	350	-	**0** (0 to 0)	⊕⊕◯◯ Low[a,b]	
**lung function (FEV1/FVC)**												
5	randomised trials	serious[c]	not serious	not serious	serious[d]	none	70	65	-	**0** (0 to 0)	⊕⊕◯◯ Low[c,d]	
**Lung function (RV/TLC)**												
3	randomised trials	serious[e]	not serious	not serious	serious[f]	none	87	77	-	**0** (0 to 0)	⊕⊕◯◯ Low[e,f]	
**Exercise capacity (6MWT)**												
6	randomised trials	serious[a]	serious[g]	not serious	serious[h]	none	104	103	-	**0** (0 to 0)	⊕◯◯◯ Very low[a,g,h]	
**Exercise capacity (VO2)**												
11	randomised trials	serious[a]	serious[i]	not serious	not serious	none	262	267	-	**0** (0 to 0)	⊕⊕◯◯ Low[a,i]	
**Exercise capacity (W máximo)**												
7	randomised trials	serious[a]	not serious[h]	not serious	serious[f]	none	170	151	-	**0** (0 to 0)	⊕⊕◯◯ Low[a,f,h]	

CI: confidence interval

Explanations

a. The certainty of the evidence was downgraded by one level due to serious risk of bias. A considerable number of included studies showed a high risk of bias in critical domains such as blinding of participants and personnel (performance bias) and blinding of outcome assessment (detection bias). In addition, several studies presented an unclear risk regarding random sequence generation and allocation concealment, which limits confidence in the estimated effects
b. The confidence interval is wide, crosses the null value (0), and includes both possible benefits and harms. The point estimate is very small and unlikely to be clinically important. Although the overall sample size is not critically small, it is insufficient to provide a precise estimate of the true effect, particularly when the effect size is so close to zer
c. Downgraded by one level for risk of bias, as several included studies show high risk in key domains such as blinding of personnel and participants, which could have influenced treatment responses or outcome measurement. In addition, at least one study presents unclear risk regarding random sequence generation and allocation concealment, raising further concerns about the internal validity of the results.
d. Downgraded by one level for imprecision, as all five included studies have confidence intervals that cross the null value, indicating uncertainty regarding the direction and magnitude of the effect. This consistent lack of precision across studies limits the

certainty that a true and clinically meaningful effect exists.
e. Downgraded by one level for risk of bias. Two studies have high risk in key domains such as blinding of participants, personnel, and outcome assessors, which could have influenced treatment perception and outcome measurement. Additionally, one study presents unclear risk in two important methodological domains, raising further concerns about the internal validity of the findings.
f. Downgraded by one level for inconsistency, as all three studies have confidence intervals that cross the null value, indicating substantial variability in the direction and magnitude of the effect. This inconsistency limits the ability to draw a clear conclusion about the intervention's effect.
g. Downgraded by one level for inconsistency, as all three included studies have confidence intervals crossing the null value and show effects in different directions. Moreover, statistical heterogeneity is high ($I^2 = 83\%$), indicating substantial variability not due to chance. This combination of high heterogeneity and inconsistency reduces confidence in the pooled effect estimate.
h. The evidence shows high imprecision. Of the six studies evaluated, five cross the null value in their confidence intervals, indicating that there is insufficient evidence to establish a clear or consistent effect. Only one study presents a narrow confidence interval that does not cross the null value, suggesting a more precise estimate of the effect.
i. The inconsistency is high (92%), indicating significant variability between the studies. This high inconsistency reflects substantial differences in the reported effects, making it difficult to interpret and generalize the results. The included studies show heterogeneous results due to variations in study designs, populations, and measurement methods, contributing to the lack of a consistent effect estimate. This reduces confidence in the overall results.
